# Genetic approaches to exploit landraces for improvement of *Triticum turgidum* ssp. *durum* in the age of climate change

**DOI:** 10.3389/fpls.2023.1101271

**Published:** 2023-01-27

**Authors:** Chiara Broccanello, Diana Bellin, Giovanni DalCorso, Antonella Furini, Francesca Taranto

**Affiliations:** ^1^ Department of Biotechnology, University of Verona, Verona, Italy; ^2^ Institute of Biosciences and Bioresources, (CNR-IBBR), Bari, Italy

**Keywords:** *triticum turgidum* ssp. durum, landraces, genotyping, breeding, molecular markers, climate change, abiotic stress

## Abstract

Addressing the challenges of climate change and durum wheat production is becoming an important driver for food and nutrition security in the Mediterranean area, where are located the major producing countries (Italy, Spain, France, Greece, Morocco, Algeria, Tunisia, Turkey, and Syria). One of the emergent strategies, to cope with durum wheat adaptation, is the exploration and exploitation of the existing genetic variability in landrace populations. In this context, this review aims to highlight the important role of durum wheat landraces as a useful genetic resource to improve the sustainability of Mediterranean agroecosystems, with a focus on adaptation to environmental stresses. We described the most recent molecular techniques and statistical approaches suitable for the identification of beneficial genes/alleles related to the most important traits in landraces and the development of molecular markers for marker-assisted selection. Finally, we outline the state of the art about landraces genetic diversity and signature of selection, already identified from these accessions, for adaptability to the environment.

## Introduction

Durum wheat (DW) (*Triticum turgidum* L. ssp. *durum*) (Desf.) is the 10^th^ most cultivated cereal worldwide, with a total production of about 38 million tons (Xynias et al., 2020). DW is grown primarily in the Mediterranean basin, accounting for 75% of global production, supported mainly by Italy, Spain, France, Greece, Morocco, Algeria, Tunisia, Turkey and Syria (**Table 1**). In addition, outside the Mediterranean basin, the major producers are Canada, Mexico, the USA, Russia, Kazakhstan, Azerbaijan, and India (De Vita and Taranto, 2019; Martínez-Moreno et al., 2022). Although DW production constitutes only 7% of total wheat production, the rest is produced from bread wheat (*Triticum aestivum*), its importance for the countries of the Mediterranean basin is pivotal. DW is considered a staple food as it constitutes the dominant part of the diet for many populations in this area. The main products derived from DW include pasta, cous cous, burghul, and bakery products. Durum wheat-based products have a low glycemic index which makes them healthy products that can be recommended for low-carb diets (Di Pede et al., 2021). Furthermore, DW constitutes the main food source for 1.2 billion poor people, providing 20/50% of daily calories, 20% of protein, and is considered a strategic crop for food security. Regarding the economic importance of DW, Italy is the world’s largest producer of pasta with over 3.36 million tons/year of pasta produced, and the leading country for exports with 1.9 million tons/year (Altamore et al., 2020). On a cultural level, DW and its ancestor wild emmer (*Triticum turgidum* ssp. *dicoccoides*) have been at the foundations of food, from the Neolithic period to the Greeks and the Roman Empire, up to the present day (Martínez-Moreno et al., 2020). Its cultivation and processing constitutes a cultural heritage.

**Table 1:** Durum wheat of the major producer countries in European Union: area, production, yield and growing seasons (source: DG Agriculture and Rural Development based on Eurostat crop production annual data).

Country	Durum wheat area (thousand hectares)			Durum wheat production (thousand tonnes)			Durum wheat yield (tonnes/hectare)			Growing seasons
	2019	2020	2021	2019	2020	2021	2019	2020	2021	
**EU**	**2,145**	**2,112**	**2,206**	**7,476**	**7,420**	**7,822**	**3**	**4**	**4**	
Italy	1,224	1,210	1,229	3,849	3,885	4,065	3	3	3	July and August
Spain	267	251	298	704	787	744	3	3	2	June and July
Greece	254	263	220	684	794	573	3	3	3	From June to August
France	246	252	294	1,566	1,321	1,581	6	5	5	From June to August
Slovakia	44	34	49	188	174	292	4	5	6	From June to August
Hungary	37	27	30	162	121	160	4	4	5	From June to August
Germany	32	34	38	155	183	207	5	5	6	From June to August
Austria	17	17	19	81	79	88	5	5	5	From June to August
**Others EU**	26	25	29	86	75	113	n.a.	n.a.	n.a.	

However, the on-going climate change threatens DW production and is subjecting this crop to stresses rarely experienced. In the Mediterranean area and western Europe, climate projections for the 2040-2070 interval warn that extreme drought events will become more frequent and severe due to decreased winter precipitation and increasingly dry springs (Spinoni et al., 2018). In a recent study, it was estimated that global warming may reduce the world’s suitable areas for DW cultivation by 19% in 2050 and by 48% in 2100, with the greatest losses occurring in the Mediterranean basin which is recognized as a climate change hotspot (Shayanmehr et al., 2020; Martínez-Moreno et al., 2022). The main environmental constraints influencing the yield of DW in this area are drought, high temperatures, and salinity (De Vita and Taranto, 2019). These stresses, if occurring in growth stages such as flowering, pollination, and grain-filling, can strongly affect crop productivity.

**Figure 1:**
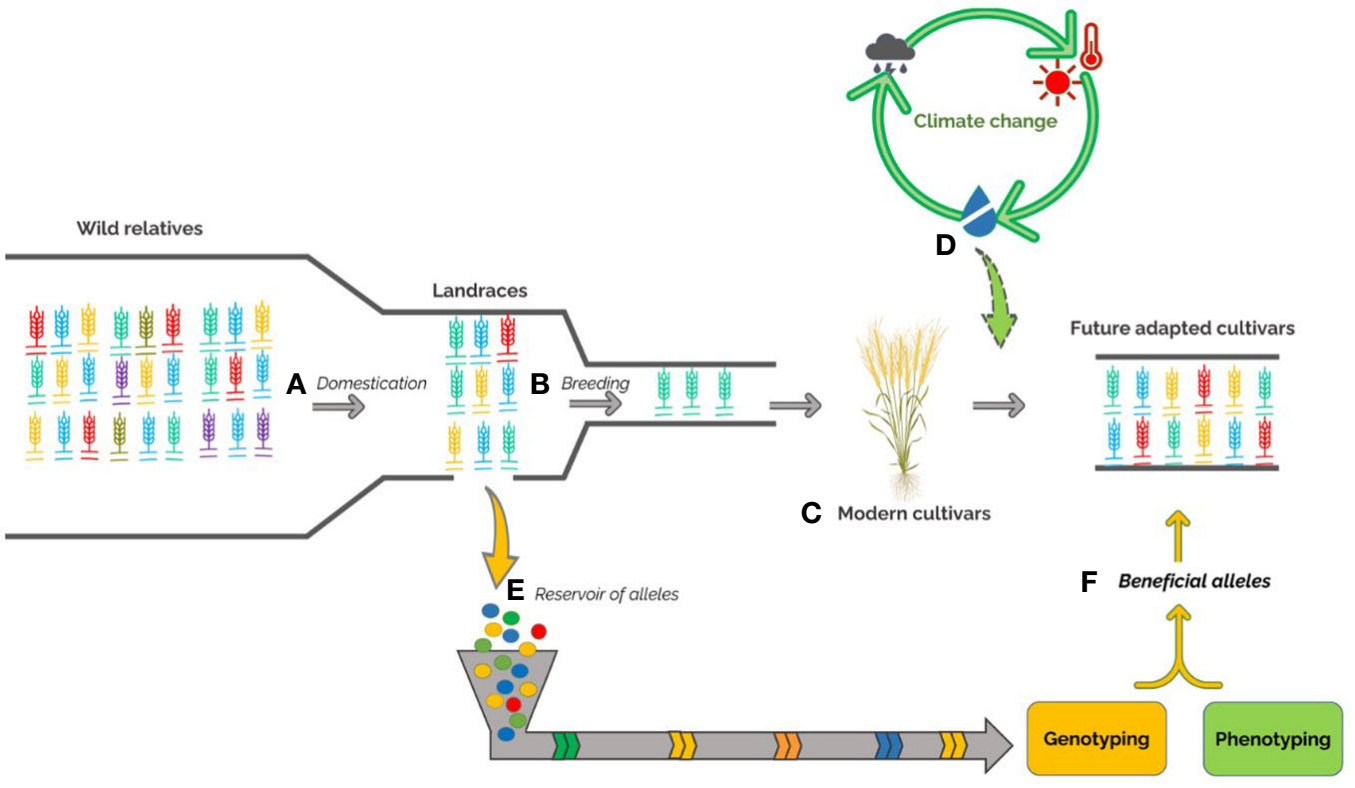
Development of durum wheat over time, including the loss of the diversity through the genetic bottlenecks of domestication **(A)** from wild relatives to the selection of landraces and plant breeding activities **(B)** moving from landraces to modern cultivars **(C)**. The emergence of climate change **(D)** requires to broad the genetic basis of modern cultivars. The exploration and exploitation of genetic variability within landrace germplasm **(E)** coupled to -omics approaches will be useful to discover beneficial alleles **(F)** and develop new modern cultivars best adapted to environmental changes.

This review aims to highlight the important role of durum wheat landraces as a useful genetic resource to improve the sustainability of Mediterranean agroecosystems, with a focus on adaptation to environmental stresses (**Figure 1**). We described the most recent molecular techniques and statistical approaches suitable for the identification of beneficial genes/alleles related to the most important traits in landraces and the development of molecular markers for marker-assisted selection. Finally, we outline the state of the art about landraces genetic diversity and signature of selection, already identified from these accessions, for adaptability to the environment.

## Durum wheat cultivation: An historical overview

Durum wheat (tetraploid, genome AABB, 2n=4x=48) is a cereal grain evolved from the tetraploid domesticated emmer wheat *Triticum turgidum* ssp. *dicoccum* (Schrank ex Schübl.) Thell (Özkan et al., 2002). Domestication of wild emmer (*Triticum turgidum* L. ssp. *dicoccoides*) occurred in the Fertile Crescent (Israel, Jordan, Lebanon, Syria, south-eastern Turkey, northern Iraq, and western Iran) about 8000 years BCE (Before Common Era) from limited founder lineages (Özkan et al., 2002; Wang et al., 2022). Emmer wheat has been the main cereal together with einkorn and barley during the Neolithic period and the Bronze age. Then, it was progressively replaced by the tetraploid naked DW, spread by land through the Balkans and the maritime routes to the Mediterranean regions of Southern Italy, France, Spain, and Greece (Martínez-Moreno et al., 2020). Finally, DW became a prominent crop at about 300 BCE during the Hellenistic period. DW was commonly cultivated in the Roman Republic where the writers started to call it *Triticum* in Latin. Later, the early Islamic world and then the Arab empire further promoted the spread of the cultivation of DW through the Mediterranean areas by introducing several food types based on semolina (dry pasta and couscous). Until 1950-1955 most of the DW Mediterranean areas were cultivated with DW landraces, local accessions adapted to their place of origin (Martínez-Moreno et al., 2020). However, the first breeding activities were started in Italy in the early 1900s, resulting in the release of the most renowned cultivar Senatore Cappelli in 1915, by the breeder Nazareno Strampelli (Scarascia Mugnozza, 2005). Since then, the cv. Senatore Cappelli appeared in the pedigree of almost all new DW varieties due to its repeated use in DW programs until the end of the 1960s (Laidò et al., 2013). Afterward, the introduction of the law n. 580/67 for Pasta Pureness gave the impulse to start the development of private seed companies and international research centers, such as CIMMYT and ICARDA. In particular, the development of the CIMMYT-derived semi-dwarf wheat varieties led to the Green Revolution in several countries. During the period between 1960’-90’, breeding activities coupled with mutagenesis generated new genetic variability (Xynias et al., 2020) and efforts were made to release every year improved varieties with high yield potential and other interesting traits. Such more productive cultivars were preferred for cultivation over large areas. Therefore, the multitude of DW landraces planted for centuries was progressively replaced. This replacement has led to an important erosion of the environmental adaptation traits evolved during the years by the landraces.

## Environmental challenges for durum wheat cultivation

The climate characteristics of the Mediterranean region have played a significant role in shaping the phenotypic (and the genotypic) configuration of both DW wild relatives and cultivated varieties. This basin is characterized by hot and dry summers, followed by cold and wet winters. Climate change, particularly important in the last decades, points to an increased variability, in which drought events, often coupled with heat waves, can hamper growth and development, eventually affecting crop yield. For example, yield is reduced of about 5% per Celsius degree of increase in temperature, with a gross loss reaching 24% under a growth temperature of 31°C during flowering (Liu et al., 2016). Clearly, the negative effect of the abiotic stress depends on its duration and the phenological phase of the plant. For instance, sudden and extremely high temperature (T > 32°C) for a short duration (3 to 5 days) is referred to as a *heat shock*, while moderately high maximum temperature (20 to 30°C) for a longer duration is known as *chronic heat stress* (Li et al., 2013). In DW, the most sensitive stages to heat stress are anthesis and grain filling (Chaparro-Encinas et al., 2021). Heat stress alters membrane fluidity and enzyme activity which in turn hamper respiration and photosynthesis, and related processes (e.g. electron flow and carbon fixation metabolism, starch accumulation and stability, architecture and functioning of thylakoids), as well as water assimilation and nutrient absorption and allocation in the plant body. After phase transition, this results in compromised pollen viability, aberrant macrosporogenesis, starch synthesis, and grain filling, responsible for the reduction in yield. Reduced water availability, due to both erratic or deficient rainfall and limited irrigation, worsens the negative effect of heat stress, hindering grain yield (in terms of seed number and weight) and technological quality and protein composition (Flagella et al., 2010). Drought stress is induced also by soil physical characteristics, which significantly affect water holding capacity and supply, influencing water and nutrient absorption by roots. Plants respond to drought and heat stress by enacting similar physiological mechanisms. Transcriptome analysis of heat susceptible and tolerant wheat revealed the involvement of multiple processes associated with tolerance to heat shock and drought stress, including the formation of deeper roots, synthesis of heat shock proteins, stomatal control, coordination of transpiration rate, and enhancement of osmoprotective response (Kulkarni et al., 2017). Also, the use of genome wide mapping approaches is providing abundant information about genomic regions associated to heat tolerance (Sukumaran et al., 2018).

Soil geo-biochemistry, geographical localization (sea proximity, with seawater intrusion into freshwater aquifers), and events of rising groundwater table can increase the amount of salts in soils. Moreover, anthropogenic activities, such as inappropriate irrigation and drainage practices or irrigation with brackish water, determine salt accumulation in the soil surface or within the solum, causing salinity stress in plants (Annunziata et al., 2017). Soil salinity is usually referred to the increased amount of Na^+^/Cl^¯^ in the soil upper layer, but a variety of ions, mainly K^+^, Ca^2+^, Mg^2+^, and ${\text{SO}}_{4}^{2-}$ , ${\text{HCO}}_{3}^{-}$ , ${\text{NO}}_{3}^{-}$ , can also accumulate. On one hand, salinity leads to permanent modifications of the soil structure by decreasing soil aeration, leaching, and infiltration rate, and increasing runoff and soil erosion (Edelstein et al., 2010). On the other hand, salinity affects plant physiology and growth. Stress effects harbored by salinity are usually due to both (*i*) cell toxicity of the accumulated ions, which often results in nutrient imbalance and enhanced oxidative stress, and (*ii*) osmotic stress, due to the extremely low water potential of soil along with a reduction in water assimilation. Therefore, cellular, and metabolic processes involved to counteract salt stress are comparable to those induced by drought (Munns et al., 2006). Interestingly, DW is conventionally considered a tolerant crop enduring up to 5.9 dS/m (De Santis et al., 2021). In field conditions with 10 dS/m NaCl, DW produced a reduced yield, compared with rice that died before maturity (Munns et al., 2006). Lower level of salinity reduces the leaf area and shoot biomass, while grain yield is not affected. Tolerance to salinity is associated with low rate of Na^+^ root-to-shoot transport and higher selectivity for K^+^ than for Na^+^. Indeed, a correlation between grain yield and Na^+^ exclusion from the vegetative organs, together with the enhanced K^+^/Na^+^ discrimination in root absorption and xylem loading has been identified in tolerant genotypes (Munns et al., 2000), which confirmed xylem transport, as one of the main discriminants between sensitive and tolerant species (Davenport et al., 2005). Tolerance to salinity is a quantitative trait controlled by many genes. Moreover, it appears that the expression of genes responsible for salt tolerance depends on plant age and ontogeny. Also, environmental factors contribute to the large phenotypic variation reported, enhancing the difficulty of breeding programs aimed to improve salt tolerance (De Santis et al., 2021). In wheat, a QTL mapping approach has identified the locus Nax1 (involved in limiting Na^+^ translocation to the above-ground tissues and inducing salt tolerance), mapped to the long arm of chromosome 2A, responsible for almost 38% of phenotypic variation in low Na accumulation in the mapping population, and this locus, together with closely linked markers, are commonly adopted to select salt tolerant durum genotypes (Lindsay et al., 2004). Other characteristics of salt-tolerant genotypes include differential ion partitioning between aged and young leaves, cell osmotic adjustment contrasting osmotic stress, and early phase-transition, leading to a shorter growing season (Colmer et al., 2005).

Drought, heat, and salt stress, being linked to each other, induce the generation of reactive oxygen species (ROS), including hydrogen peroxide, superoxide, or hydroxyl radicals, which are continuously formed mainly in the cytosol, chloroplasts, and mitochondria (Laus et al., 2022). ROS have a significant role in signaling but, under stress conditions, their over-accumulation may be responsible for the oxidative stress characterized by membrane peroxidation, protein degradation, and DNA mutation, eventually leading to the death of the plant cell. Plant cells are usually equipped with a great variety of ROS scavenging enzymes including superoxide dismutase, catalase, and glutathione peroxidase, and antioxidants, such as ascorbic acid, tocopherol or glutathione, which also contribute to ROS detoxification (Dvorak et al., 2021). Interestingly, the tolerance of DW genotypes to environmental stresses leading to ROS production has been widely associated with higher activities of scavenging enzymes, pointing to a role of these mechanisms in the drought and salt tolerance in particular genotypes (Laus et al., 2022). Therefore, they are a good candidate to be considered in DW breeding programs. Anyway, it should be stressed that as the DW sensitivity to stress is influenced by the phenology, also the antioxidant performance depends on the stress characteristics (severity and duration), on the stage of development at which the stress acts, and on the plant organs targeted. Finally, (as shown by the increasing literature on the topic, Li et al., 2013; De Santis et al., 2021), breeding programs should also keep the attention on the stress effect on quality traits of DW grains. Indeed, changes in grain content and composition, affecting technological and health quality (e.g. protein, starch and dietary fiber accumulation and composition, phytochemical, and health-related micronutrient accumulation), are incredibly susceptible to environmental clues and stress and must be taken into account when implementing the breeding programs.

## Durum wheat landraces: An endless treasure

Many efforts are made by researchers and breeders to constantly look for new sources of genetic variability to improve the elite varieties for adaptation traits, to face climate change. The exploitation of the existing genetic variability, still available in landraces, is one of the best approaches to adopt. According to Camacho Villa et al. (2005), a landrace is “a dynamic population of a cultivated plant that has a historical origin, distinct identity, and lacks formal crop improvement, as well as often being genetically diverse, locally adapted, and associated with traditional farming systems”. It is the result of natural and/or farmer-mediated evolutionary forces that generated plants better adapted to the local climate/environmental conditions (Zeven, 1999).

They are considered a reservoir of useful alleles that can be exploited to broaden the genetic basis of important adaptation traits. Landraces are rich in micronutrients and have high concentrations in total phenol and antioxidant content, as well as in tocols, carotenoids, and lutein (Azeez et al., 2018). Since the landraces can tolerate abiotic and biotic stresses, their yield is lower than modern varieties (Tan, 2002). For this reason, landraces are no longer cultivated over large areas where the more productive cultivars are preferred. Anyhow, several landraces have been rediscovered and reused thanks to their adaptation to sustainable and low-input cropping systems. They produce a great amount of straw, which, when used for animals, can make them economically more convenient than modern varieties, or preferable for organic farming because of their greater competitive ability against weeds (Lemerle et al., 2001; Annicchiarico et al., 2005).

**Figure 2:**
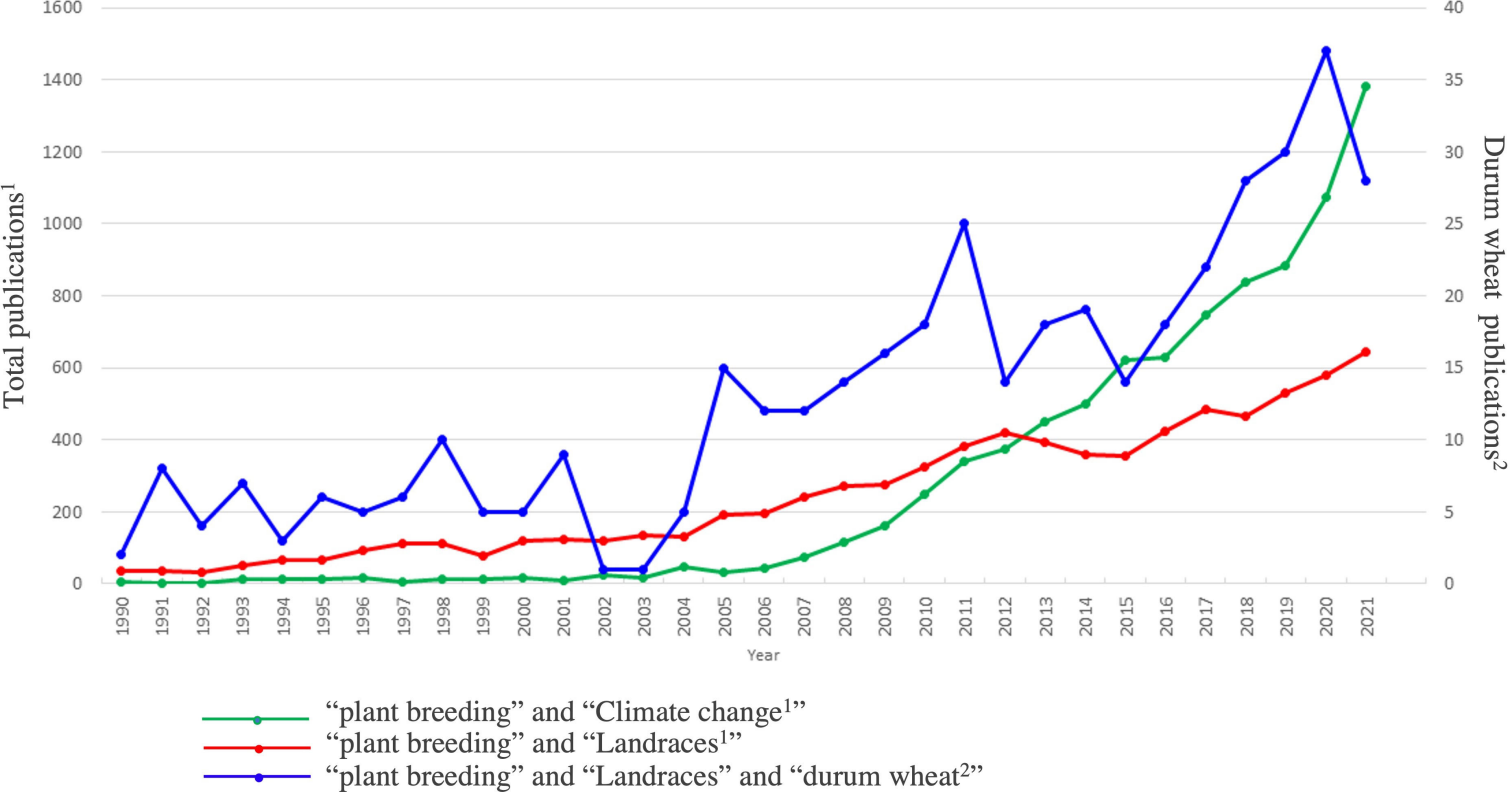
Number of publications in which climate change, landraces, and durum wheat are associated to plant breeding. The scale of the primary vertical axis shows the values for the associated data series in green: complete query = (“plant breeding” AND “climate change”) and red: complete query = (“plant breeding” AND “landraces”); the scale of the secondary vertical axis shows the values for the associated data series in blue: (“plant breeding” AND “landraces” AND “durum wheat”). The analysis is based on the information available in the Web of Science database (www.webofknowledge.com), category “Plant science”, considering the time interval of 1990–2021. Different keywords (i.e., “plant breeding”, “landraces”, “climate change” and “durum wheat”) and Boolean operators were used to query the database.

Indeed, thanks to the efforts of farmers and scientists, wheat landraces and old cultivars have been collected and conserved by *in-situ* or ex-situ strategies. The *in-situ* strategy relies on individual farmers who traditionally cultivate landraces for their production or are sponsored by the government or private companies. The ex-situ conservation is managed by international institutions such as CIMMYT, ICARDA, and USDA or by national projects led by local universities (Adhikari et al., 2022). With the advance of modern technologies, phenotyping and genotyping are extremely affordable, and the landraces can be studied both for their conservation and for molecular markers development (Nazco et al., 2012; Marone et al., 2021). The exploration of genetic variability in landrace germplasm has become an issue of great global interest, mainly during the last two decades. **Figure 2** shows how the number of publications related both to “plant breeding” and “landraces”, and “plant breeding” and “climate change”, have had a strong increase since 2005, when “The 2005 United Nations Climate Change Conference” took place and marked the entry into force of the Kyoto Protocol. From 2005 to 2021 the number of publications related to the plant breeding strategies, to cope with climate change, has grown exponentially. Also for “durum wheat” and “landraces”, it can be noted a similar trend, although the number of publications is scarce. Some works aimed to characterize specific DW landraces are reported in **Table 2**, which includes a list of the most important European landraces specially studied both for stresses and quality related traits.

**Table 2:** List of the most important durum wheat landraces.

Country	Common name/Accession	Trait	References
Italy	Tumminia SG3	polyphenols profile and content	Lo Bianco et al., 2017
Italy	Scavuzza	polyphenols profile and content	Lo Bianco et al., 2017
Italy	Russello SG8	polyphenols profile and content	Lo Bianco et al., 2017
Italy	Ruscia	polyphenols profile and content	Lo Bianco et al., 2017
Italy	Manto di Maria	polyphenols profile and content	Lo Bianco et al., 2017
Italy	Margherito	polyphenols profile and content	Lo Bianco et al., 2017
Italy	Biancuccia	polyphenols profile and content	Lo Bianco et al., 2017
Italy	Bidì	suitable characteristics for malting and brewing	Alfeo et al., 2018
Italy	Francesa	suitable characteristics for malting and brewing	Alfeo et al., 2018
Italy	Gioia	suitable characteristics for malting and brewing	Alfeo et al., 2018
Italy	Giustalisa	suitable characteristics for malting and brewing	Alfeo et al., 2018
Italy	Inglesa	suitable characteristics for malting and brewing	Alfeo et al., 2018
Italy	Martinella	suitable characteristics for malting and brewing	Alfeo et al., 2018
Italy	Bufala Bianca	malt charaterisrics suitable for brewing	Alfeo et al., 2021
Italy	Bufala nera corta	malt charaterisrics suitable for brewing	Alfeo et al., 2021
Italy	Bufala rossa lunga	malt charaterisrics suitable for brewing	Alfeo et al., 2021
Italy	Russello	high content of antioxidant phenolic compounds	Visioli et al., 2021
Italy	Trentino	suitable characteristics for malting and brewing; polyphenols profile and content	Alfeo et al., 2018; Lo Bianco et al., 2017
Italy	Tripolino	polyphenols profile and content	Alfeo et al., 2018; Lo Bianco et al., 2017
Italy	Urria	polyphenols profile and content	Alfeo et al., 2018; Lo Bianco et al., 2017
Italy	Timilia	high content of antioxidant phenolic compounds	Taranto et al., 2022
Portugal	PI 192051	stem rust resistance sources	Aoun et al., 2019
Portugal	Aus26582	leaf rust resistance	Qureshi et al., 2018
Portugal	Aus26579	leaf rust resistance	Qureshi et al., 2018
Cyprus	IG-82549	glutenin protein composition	Moragues et al., 2006
Portugal	Lobeiro de Grao Escuro	having high EU quality index and high protein quality	Nazco et al., 2014a
France	Trigo Glutinoso	having high EU quality index and a high sedimentation index	Nazco et al., 2014b
Spain	BGE-013614	glutenin protein composition	Moragues et al., 2006
Spain	BGE018675	higher zeaxanthin relative content	Requena-Ramírez et al., 2021
Spain	BGE045643	higher zeaxanthin relative content	Requena-Ramírez et al., 2021
Spain	BGE045657	higher zeaxanthin relative content	Requena-Ramírez et al., 2021
Spain	BGE018321	higher relative β-carotene content	Requena-Ramírez et al., 2021
Spain	BGE045628	higher relative β-carotene content	Requena-Ramírez et al., 2021
Spain	BGE045633	higher relative β-carotene content	Requena-Ramírez et al., 2021
Spain	BGE030921	highest α-carotene content	Requena-Ramírez et al., 2021

Because of the genetically heterogeneous nature of landraces, which are in a constant state of evolution due to different factors such as ecogeographical area and conventional or modern breeding techniques (Casañas et al., 2017), the establishment of core collections represents an affordable cost approach to reduce the degree of co-ancestry redundancy and the genetic stratification in the landraces whole collections. The goal of creating core collections is to maximize the allelic richness at molecular markers and best represent variation at phenotypic traits, in order to define the smallest possible number of individuals that represents a more compact and manageable population. Core collections were made for Spanish, Indian, Iranian, and Israeli-Palestinian landraces (Etminan et al., 2017; Ruiz et al., 2012 and 2013; Frankin et al., 2021; Phogat et al., 2019; Chacón et al., 2020). In addition, a core set of landraces was developed starting from the Global Panel of Durum Wheat (GPD), reducing their number from 416 to 192 (Mazzucotelli et al., 2020 and Kabbaj et al., 2017). This approach is useful not only to represent whole genetic diversity but also to enquire and identify new sources for interesting traits. For example, SNP markers associated to resistance to leaf rust, tan spot and *Stagonospora nodorum* blotch were identified using the core collection created from the Watkins collection (Martínez-Moreno et al., 2021, Halder et al., 2019). Moreover, the core collections have also been used for unlocking the genetic and morpho-physiological adaptation traits to semi-arid environments (Abu-Zaitoun et al., 2018) and to study agronomic and quality traits, such as root architecture, stem cross section, height, heading date and carotenoid content (Ruiz et al., 2018; Ávila et al., 2021; Requena-Ramírez et al., 2021).

## Durum wheat genome and pangenome assemblies

The DW sequencing project has been carried out by Maccaferri et al. (2019) using the modern cultivar Svevo. The annotation led to the identification of 66,559 genes, where the gene density distribution reflects the QTL density distribution. The comparison between wild emmer Zavitan and Svevo genomes identified putative loci under domestication and selection, and localized the reduction in diversity mainly in the pericentromeric regions of the chromosomes (Maccaferri et al., 2019). However, in the evolution of DW landraces, the reduction of diversity was more spread along the genome as a consequence of breeding programs.

Resequencing techniques, such as whole genome resequencing, are not suitable for species with complex genomes, for which a reduction of genomic complexity prior to NGS-based SNP discovery is preferred (Borrill et al., 2019). In polyploid species such as DW, with a large genome size (about 10.45Gb) and a large proportion of repetitive sequences (> 85%), the presence of paralogous and multi-copy sequences adds complexity in classifying the correct scoring of SNPs at a single locus for SNP discovery (Sandve et al., 2010).

In the last decades, the number of sequenced genomes in crop species has continued to increase exponentially, highlighting the presence of large structural variations between individuals of the same species. Therefore, relying on the single reference genome cannot represent the entire sequence diversity present in a population (Golicz et al., 2016). This observation led to the concept of “pangenome”, that describes the landscape of genetic variation within a species, in order to capture a comprehensive view of genetic diversity that include the entire crop gene pool (Bayer et al., 2020; Khan et al., 2020). In the pangenome development, it is pivotal to consider the genetic variation brought by the crop wild relatives, in order to include as much genetic variability as possible (Khan et al., 2020). In fact, wild species preserve important genes related to tolerance to various types of stress that were lost during the domestication process. The increasingly less expensive sequencing approaches have allowed to deepen the genetic architecture of the crop wild relatives leading, in the past decade, to several *de novo* sequencing projects developed also in crop wild relatives. In soybean, 14 cultivated and 17 wild accessions were resequenced, confirming the great allele diversity present in wild accessions (Zhou et al., 2015). In maize, 75 lines including cultivated, wild and landrace accessions were resequenced, highlighting the genes linked to selection and providing evidence for introgression from wild relatives (Hufford et al., 2012).

In wheat, the first pangenome has been constructed using the bread cv. Chinese Spring as suitable reference assembly, followed by the expansion of this reference with 16 additional sequences from other bread wheat varieties (Montenegro et al., 2017). Graph pangenomes based on 16 public assemblies (Wheat Panache) was developed with the aim to discover genome variation between cultivars and to mine the diversity present in the large and complex wheat genome (Bayer et al., 2022). However, the mathematical modeling of pangenome expansion revealed that all these wheat varieties have a closed pangenome; therefore, the inclusion of more distant accessions such as wild relatives and landraces could harbor yet unexplored sequence variants that may further increase the gene content of the pangenome. The use of divergent individuals may increase the number of novel gene variants as well as improve the accuracy of the read mapping for SNP discovery. The use of landraces can support the breeding of cultivars better adapted to diverse environments and more resilient to climate change; indeed, plant pangenome assemblies have shown that genetic variations are often associated with biotic or abiotic stress.

No pangenome has yet been assembled for durum wheat, although several projects are underway. Indeed, the use of Svevo genome as suitable reference may accelerate the sequencing of new durum cultivars enabling the pangenome construction. As far as is known, at the moment the only reference genome for durum wheat remains Svevo. Given the growing interest in some European landraces (**Table 2**), the release of new genome assemblies from landraces is expected in the next few years.

## High-throughput genotyping techniques

### Exome and RNA sequencing

A widely employed method for *de novo* SNP discovery and genotyping in large genome-size species is the exome sequencing. The workflow involves the fragmentation of high-quality genomic DNA, repair ends, adenylation, adapter ligation, and the selective hybridization of probes for target enrichment. Then two consecutive captures of the hybridization probes ensure high specificity of target region before the sequencing step (Kaur and Gaikwad, 2017). Ready to use exome kits and their customization are available for many crop species such as wheat (Harrington et al., 2019). Exome sequencing techniques have been used to identify polymorphisms and genes in the tetraploid wheat genome, also in combination with bulk segregant analysis (Saintenac et al., 2011; Mo et al., 2018). In the last few years, thanks to the release of wheat reference genomes and annotations, this approach is increasingly used.

An alternative to exome capture is the high-throughput RNA sequencing which analyzes sequence variations in the transcribed portion of the genome. RNA-seq is the technique of choice for the identification of new genes and isoforms, and for the detection of variants including expressed SNPs and INDELs. Furthermore, this approach allows the identification of differentially expressed genes in plants under stress conditions. However, this technique is expensive since the reagents for the sequencing of the entire transcriptome are required. To overcome this problem, a new technique that is emerging also in plants (it is already widely used in human genetics) is the target RNA sequencing. This technique allows very high precision in the discovery and quantification of genes because it sequences only those of interest. The most important step of target RNA-seq is the design of the specific probes that can be customized to meet the specific needs of each experiment. Since only the genes of interest are sequenced, the coverage can also be very high, allowing to increase the power to assemble low expression transcripts (Ostezan et al., 2021).

### Reduced representation sequencing (RRS)

With the decrease in the NGS cost, sequencing techniques are increasingly used as genotyping tools. However, to afford sequencing in large genome size species, reduced representation sequencing (RRS) approaches can be considered (Davey et al., 2011). Genotyping-by Sequencing (GBS) is the most used technique to greatly reduce genome complexity using restriction enzyme(s) digestion (Elshire et al., 2011; Rasheed and Xia, 2019). The digestion occurs in the presence of a specific combination of enzymes recognizing rare-rare, frequent-rare, or frequent-frequent restriction sites. In wheat, since the complexity of the genome is very high, it is normally used a double enzyme digestion (Poland et al., 2012). The digested DNA is then ligated with adapters, amplified through PCR, and sequenced. The generated data are directly used for genotyping (Deschamps et al., 2012). Typically, the sequencing involves 100-150 bp. This technique has a simple protocol, specific and reproducible, with a reduced sample handling, without the need of a reference genome (Davey et al., 2011). These properties make GBS a genotyping technique suitable for a number of species and genetic studies (Chung et al., 2017), such as genomic selection (Poland et al., 2012), SNP marker development (Forrest et al., 2014), and genetic diversity (Alipour et al., 2017). Diversity Array Technology (DArT), developed by Diversity Arrays Technology Pty Ltd. (Canberra, Australia) originally with the microarray technology platform, is one of the GBS-based techniques widely adopted in wheat, thanks to its versatility, accuracy, and low cost (Colasuonno et al., 2021).

### SNP Array

NGS technology has also created the basis for the establishment of high-density SNP arrays and the related high-throughput platforms capable of genotyping large numbers of samples (Ganal et al., 2012).

Currently, the most widely used genotyping platforms for large scale genotyping are the Infinium platform from Illumina (San Diego, CA, USA) and the Axiom technology from Thermo Fisher Scientific (Waltham, MA, USA) (Scheben et al., 2018). Technically, the Illumina technology is based on spheres covered with specific oligos adapted to the microwells and the amplification takes place on a single-base two-color extension with a single probe SNP marker (Steemers and Gunderson 2007). On the other side, the GeneChip^®^ array of Affymetrix uses photolithographic oligos on an array and the target SNP amplification involves assays with 30-mer probes (Thomson, 2014).

In wheat, the first SNP array developed was the 9K Infinium SNP Array by Cavanagh et al. (2013) used for genotyping 2,994 lines of bread wheat. Later, the array with 90K SNPs was fine-tuned (Wang et al., 2014). However, both of these chip had a greater representation in cultivated varieties, thus their use was very limited in the study of landraces (Rasheed et al., 2018). This problem was overcome by the development of the 820K Affymetrix Axiom SNP array which relied on exomes sequencing of 43 bread wheat and wild species accessions (Winfield et al., 2016). Axiom 35K SNP was then developed from this array, capable of analyzing even wild accessions at a more limited cost (Allen et al., 2017). In parallel, the Chinese Academy of Agricultural Sciences (CAAS) also developed an array containing 660K SNPs (Jin et al., 2016). More recently, in 2019, Wheat 50K (Triticum TraitBreed array, Rasheed and Xia, 2019) and 15K SNP arrays were developed (Muqaddasi, 2017) containing a selection of SNPs from Wheat 35K, 90K, and 660K SNP arrays.

SNP arrays have the advantage of facilitating high-density SNP scanning, have a high call rate, and are also cost effective when there is a need to genotype a high number of markers on many samples. However, a disadvantage, is that the set of SNPs is fixed and cannot be changed; they are also developed in hexaploid wheat, thus the SNPs present in the D genome will be unknown if it is applied in DW.

### Genotyping for marker assisted breeding

Among the most competitive technologies used for marker assisted breeding, with a medium/low throughput, there are TaqMan (Applied Biosystems, Foster City, CA), KASP (Kompetitive allele specific PCR, Hoddesdon, UK), and rhAmp (Integrated DNA Technology technologies, Redwood City, CA), widely used in many plant species such as wheat and sugar beet (Broccanello et al., 2018; Ayalew et al., 2019). TaqMan chemistry is based on fluorescently-tagged, allele-specific probes detected using real-time PCR-based assays, while KASP technology adopts an endpoint fluorescence detection to discriminate tagged SNP alleles. The most recent method is rhAMP, that uses RNase H2 to activate primers after successful binding to their target site. All these chemistries are suitable for use on a variety of real-time PCR instruments with different throughputs. For example, the TaqMan assays can be applied in Real-Time PCR, but also can be used with the Open Array technology (Thermo Fisher Scientific, Carlsbad, CA) which allows the simultaneous analysis of 4 OpenArray plates, each composed of 3,072 through-holes allowing the genotyping analysis of 16, 32, 64, 128, 192, 256 SNPs at a time (Broccanello et al., 2020). Perry and Lee, (2017) developed an OpenArray plate composed of 16 SNP markers able to discriminate 47 DW varieties registered for production in Canada.

These chemistries have the advantage of being highly reproducible, sensitive, and cost effective; moreover, they can be freely customized both for the number of samples and SNPs that can be analyzed, adapting perfectly to marker assisted selection for crop improvement (Broccanello et al., 2018).

## Advancements in trait genetic dissection and breeding

### Genome wide association study (GWAS)

Genome wide association study is a powerful tool to study the genetic base of complex traits and detect relationships between phenotypic variations and the associated genetic polymorphisms (Taranto et al., 2018). The statistical methods for the analysis of associations have improved over the years, going from a classic ANOVA, that generates many false positives to the development of the mixed model framework, which increases computational speed and improves statistical power (Pavan et al., 2020; Tibbs Cortes et al., 2021). Subsequent advancement in statistical analysis methods led the association analysis of all markers simultaneously. This approach is based on Bayesian methods which are normally used in genomic prediction (Fernando and Garrick, 2013). However, the most common actual methods include TASSEL (Bradbury et al., 2007), GAPIT (Lipka et al., 2012), and GEMMA (Zhou and Stephens, 2012). 58 candidate genes associated with salt tolerance have been found, in bread wheat, performing 5 multi locus GWAS models that include mrMLM, FASTmrMLM, FASTmrEMMA, pLARmEB, and ISIS EMBLASSO (Chaurasia et al., 2020; Esposito et al., 2022). In DW genome wide association studies often involve the use of landraces to identify new causative SNPs. Moreover, the genomic regions linked to wheat blast resistance were identified in Indian genotypes with a MLM (mixed linear mode) in TASSEL. A novel GWAS approach is the environmental GWAS (envGWAS) that associates the single nucleotide polymorphisms with the geographic information system (GIS) of the original samples collection sites. In this context, the genome wide association was performed to study the local adaptation of Iranian and Pakistani bread wheat landraces using an EigenGWAS approach and a fixed and random model circulating probability unification (FarmCPU) (Hanif et al., 2021).

### Genomic selection

In a context of climate change, a technique developed to accelerate breeding procedures and speed up the selection of superior genotypes is genomic selection (GS) (Crossa et al., 2017). This statistical model uses SNP molecular markers for a genomic prediction of genotype performance. The aim of GS is to predict breeding and/or genetic values. GS uses genotypic and phenotypic data for the constitution of a training population and then the predictive equation is used to select candidates that have been genotyped but not phenotyped. GS has the advantage of being able to rapidly improve complex and low heritability traits and reduce the cost of hybrid development. This technique can also be used for less complex traits with high inheritance and, for this scenario, high genomic prediction (GP) accuracy is expected. However, when a trait is controlled by a high number of loci there are several factors influencing the prediction accuracy such as the size and genetic diversity and how distant is the training from the testing population. Moreover, for complex traits with large numbers of markers that are not in linkage disequilibrium (LD) with the QTL, GP accuracy is lower (Daetwyler et al., 2010).

In general, the statistical models developed for GP are based on single-environment assessments. However, in plant breeding the presence of a Genotype x Environment (G x E) interaction may complicate the selection of stable lines. Hence, some genomic prediction models, considering the G x E interaction, help breeder select lines with optimal overall performance across different environments and in a specific target environment. Specifically, a reaction norm model, which is an extension of the random effect Genomic Best Linear Unbiased Predictor (GBLUP) model, was developed by Jarquín et al., 2014. In this model, the main effect of lines, the main effect of environments, the main effect of markers, the main effect of pedigree, and their interactions with environments, are modeled using random covariance structures that are functions of marker or pedigree genotypes and environmental covariates (Jarquín et al., 2014). Appropriate cross-validation schemes are designed to obtain valid and unbiased estimates of the predictive ability obtainable from the developed genomic prediction models (Roberts et al., 2017). The reaction norm model has already been applied for the genomic prediction of 8,416 Mexican wheat landrace accessions and 2,403 Iranian wheat landrace accessions from the CIMMYT by Crossa et al. (2016). In this work, the authors evaluated two traits in two different environments and some heritable traits in a single optimal environment. The accuracy of the prediction for some traits such as maturity, quality traits, and grain yield and yield components was around 50-70%. The most used traits of study in genome selection experiments are related to quality improvement involving the use of different prediction models divided between parametric and non-parametric. Michel et al. (2018) proved the benefit of GS over marker assisted selection investigating the prediction of dough rheological traits in early generations and adopting the parametric RRBLUP, W-BLUP function. Genomic prediction models are routinely used in the CIMMYT spring bread wheat program since 2013 (Guzman et al., 2016). These models have also been successfully applied in genomic predictions for Fusarium head blight resistance in a DW panel (Moreno-Amores et al., 2020).

### Landscape genomics

The selective pressure of abiotic stresses often varies in space, causing the evolution of advantageous mutations under their local environment, leading that genotype to have better fitness than the same genotype originating elsewhere. This differentiation process is called local adaptation and is driven by spatially divergent natural selection (Kawecki and Ebert, 2004). To trigger local adaptation, spatially divergent selection needs to overwhelm the homogenizing effect of gene flow. The study of the genetic bases of plant adaptation is crucial for the conservation and management of wild and cultivated species. Climatic stresses require a rapid evolution of populations to quickly adapt to new conditions and avoid extinction. Furthermore, to identify the genetic basis of adaptation, it is necessary to distinguish the under-selection (adaptive) genes from the pool of neutral genes. One possible approach is landscape genomics which aims to identify the gene-environment association, in particular loci associated with certain environmental variables. The landscape genomics analyses are providing unprecedented insight into the evolutionary processes and molecular basis that govern environmental adaptation. In the context of climate change, this type of analysis investigates how the species are adapting to the various types of stress they are subjected to but also could help to identify the wild relative introgression and its contribution to local adaptation (He et al., 2019). Landscape genomics integrates molecular analyses with climatic and geographic data in which samples have been collected to identify adaptive genes (Sork et al., 2013). This association analysis can be considered a valid alternative to GWAS when working with wild accessions or landraces, as they are naturally adapted to the place of origin. Moreover, the relationship between phenotypic variation and climatic factors, in DW, has been widely studied and confirmed (Annicchiarico et al., 1995; Royo et al., 2014). Recently, landscape genomics has been applied in many species such as *Populus tricocarpa*, *Beta vulgaris* spp. *maritima*, and *Arabidopsis tahaliana.* He et al. (2019) used the landscape genomics approach to find genomic windows associated with environmental adaptation in hexaploid wheat underlining the contribution to local adaptation given by wild emmer. In addition, 93 rice landraces from sub-Saharan regions were used to study adaptation to the local environment (Meyer et al., 2016). In landscape genomics, environmental information is screened for association with genetic variations through univariate or multivariate gene-environment association (GEA) analysis (Rellstab et al., 2015; Forester et al., 2018). Many statistical models have been developed for association analysis. For example, some methods involve a logistic association model such as the Spatial Analysis Method (SAM or SAMβADA), multiple logistic regression, and Generalized Estimating Equations (GEEs). Other methods involve a linear association model such as General linear models, redundancy analysis (RDA), bayenv, Spatial Generalized Linear Mixed Model (SGLMM), Latent Factor Mixed Models (LFMMs), and GWAS mixed models (Rellstab et al., 2015). However, to make the results more reliable, it would be a good practice to compare results coming from different association models.

There are 19 bioclimatic variables that can be screened, which can be downloaded from the WorldClim database, concerning the period 1970-2000; moreover, data reporting global soil salinity layers for the years 1986, 1992, 2000, 2002, 2005, 2009, and 2016 are also available. Using this association model, it is possible to detect candidate genes associated with salinity, thanks to the historical data available on the Global Salinity Soil Map website, as it was done in *Medicago truncatula* (Guerrero et al., 2018).

The relationship between genotype and environment could be also used to predict the spatial distribution of adaptive genetic variants in future climates and the future maladaptation or genomic offset that provide a direct estimate of the expected genomic vulnerability of the species toward ongoing climate change (Capblancq et al., 2020; Cavanagh et al., 2020; Capblancq and Forester, 2021).

### Speed breeding

Recent advances in high-throughput phenotyping techniques have greatly increased the accuracy of breeding programs, having the advantage of being non-destructive and large-scale methods. However, the classic breeding programs, that have allowed the improvement of varieties, have the disadvantage of being extremely long and articulated and they take 10-15 years to release a variety. A technique that allows a rapid advancement of breeding generations is known as ‘speed breeding’. This technique allows up to 6 generations of wheat per year and involves the use of fully enclosed, controlled-environment growth chambers with the addition of supplementary lighting (Watson et al., 2018). Several protocols for rapid and high-throughput phenotyping have been developed for the characterization of several important traits related to biotic and abiotic stresses in bread wheat. In durum wheat, a protocol has been developed providing multi-trait phenotyping and trying to accelerate even more the breeding cycles by using early filial generations (Alahmad et al., 2018). These ‘speed breeding’ techniques integrate perfectly with the new technologies of high-throughput genotyping and genomic selection.

## Genetic diversity and signature of divergence in landrace germplasm

Until a few years ago, DW was well adapted to the Mediterranean environment. More recently, due to the climate crisis, drought, salinity, and low nutrient inputs occurring during flowering, pollination, and grain-filling represent the major stresses which adversely affect crop yield and quality, thus hampering agricultural productivity. Landraces coming from the Mediterranean basin are considered a particularly important group of genetic resources thanks to their high variability and tolerance to drought, pests, and adaptability to low farming systems (Lopes et al., 2015). Nowadays, the recovery, conservation, and enhancement of landraces are becoming central to increase the resilience of agricultural systems. However, how to exploit the genetic diversity of landraces to deal with environmental stress resilience is unclear and scattered (Lopes et al., 2015).

With the advance in genomic sequencing technologies and the release of the DW genome (Maccaferri et al., 2019), there has been a growing interest in comparing the patterns of genetic variation observed in landraces and modern varieties. These analyses were often focused on panels of landraces with a specific geographical origin. Population structure analysis was conducted for example in Iranian, Ethiopian, Tunisian, Turkish, and Italian germplasm revealing that, in most cases, landraces clustered separately from modern cultivars (Fayaz et al., 2019; Alemu et al., 2020; Alsaleh et al., 2022; Miazzi et al., 2022). Interestingly, a high level of genetic variation within landrace populations was detected, according to their geographical and climate of origin, revealing the importance of these factors in shaping wheat genome (Alipour et al., 2017; Taranto et al., 2022). On the other hand, cases of synonyms or homonyms as well as the presence of higher admixture of accessions between different populations of landraces were discovered, probably due to the exchange of seeds associated with human migration over time.

Examining wider collections, including landraces from different geographic origins, opens the possibility of investigating relationships on a wider global level and provides also a more precise estimation of the genetic diversity within each group. Genotyping the already mentioned core GPD by means of the iSelect 90K SNPChip followed by structure analysis showed comparatively limited genetic diversity in modern cultivar and a closer relationship to specific landrace population (North Africa and Transcaucasia). Landraces from Ethiopia appeared instead as the more isolated and distant to modern cultivars, while a high admixture level within landrace populations was confirmed (Maccaferri et al., 2019).

In addition, genome-wide population structure uncovers divergent selection during modern wheat breeding, suggesting the existence of untapped gene pools which will provide a basis for DW improvement in the next future. Many hotspots of selection were detected in the genomic regions where there are located the genes for adaptation, quality, grain yield, and stress response (Taranto et al., 2020; Soriano et al., 2021). These hotspots included important loci such as the photoperiod (*Ppd*), the vernalization (*Vrn*), and the dwarfing (*Rht*) genes, as well as loci associated with nitrogen use efficiency, plant architecture and grain yield (*TaASN3, asparagine synthetase 3*; *NR1, nitrate reductase 1*; *Fd-GOGAT, ferredoxin-dependent glutamate synthase*; *GS, glutamine synthetase*; *Sus2, sucrose synthase 2* and *TEF*, *transcript elongation factor*). In addition, genes related to quality such as pasta-making quality and color of semolina and other durum wheat-end products were also divergent between landraces and modern cultivars. In detail, loci for gluten composition (HMW/LMW, high/low molecular weight, and α, β, γ, and ώ gliadins), as well as loci involved in the carotenoid pathway (*Psy*) and polyphenol oxidase reaction (*Ppo*) were identified in hotspot regions (Requena-Ramírez et al., 2022; Taranto et al., 2022). Other divergent loci with implications in disease resistance, plant-microbe interactions, abiotic stresses, and plant development corresponded to gene models involved in important biological functions (Soriano et al., 2021). However, the identification of these genes and their allelic variants in the germplasm of indigenous DW varieties has been mainly carried out by *in-silico* analysis, at least for now. Future studies will be needed to validate the potential of the new allelic variants discovered in landraces.

## Environmental adaptation traits from durum wheat landraces

In DW, domestication and, more lately, selection and fixation of favorable alleles had led to genetic erosion, lowering the buffering capacity of modern elite cultivars towards varied climatic conditions and strongly reducing potential for improvement (Tanksley and McCouch, 1997; Lopes et al., 2015). The systematic search and discovery of genetic resources from landraces by means of genotypic and innovative phenotypic profiling of genetic resource collections and the introduction in elite crops through pre-breeding efforts are being currently implemented in bread wheat and could be a promising strategy also for DW (Reynolds et al., 2021; Sharma et al., 2021; Schulthess et al., 2022). Improvement of yield potential of landraces, by considering them as recipients, can be undertaken as an alternative strategy for developing better cultivars adapted to climate change. Inherent agronomic inferiority and disease susceptibility would hinder the direct utilization of landraces for breeding programs. However, the genomic tools and approaches we have described (i.e. genomic prediction and selection) could also strengthen pre-breeding efforts aiming at the improvement of genetic backgrounds of landraces, thereby attempting to achieve agronomic superiority starting directly from landraces as recipients and making this second alternative approach a possible and viable option (Adhikari et al., 2022). Finally, more complex breeding approaches, to develop new “synthetic” wheat crops exploiting genetic resources from wild species instead of landraces, also exist and have been already undertaken in the past (Reynolds et al., 2007; Balla et al., 2022).

Desirable genes were already identified in landraces and exploited for DW cultivars improvement by classical breeding. The most renowned DW cultivar is Cappelli, which is assumed to have been selected from the North-African landraces (De Cillis, 1942). However, the cv. Cappelli has a height of about 1.80 meters; therefore, several breeding activities were focused to create new variability by crossing the cv. Cappelli with Syriacum landraces (Aziziah, Eiti, Sinai, Tripolino). The result was the introduction of cultivars such as Capeiti 8 and Patrizio 6 which had slightly lower height, higher yield, earliness, and lodging resistance compared to Capelli, while preserving grain quality. Other similar examples of superior DW varieties, obtained by introgressing traits from landraces, were released in the frame of other breeding programs conducted in different countries (Kabbaj et al., 2017; Martínez-Moreno et al., 2020).

Molecular mapping technologies such as bulk segregant analysis (BSA), gene/QTL mapping, and genome-wide association studies (GWAS), supported by the high-throughput genotyping tools and strategies previously described, importantly increased the rate discovery of genes/QTLs regulating biotic, abiotic stress resistance, agronomic and quality traits from landraces. Several studies have been already undertaken so far to identify new genes/traits in landraces useful for breeding (see **Table 3** for a comprehensive but not exhaustive list). As can be clearly appreciated, in most recent studies traits and genes have been often also mapped by taking advantage of the newly released genomic tools for DW here described, to further support their prompt exploitation in breeding.

**Table 3:** Publication list regarding resistance/tolerance traits to biotic and abiotic stresses, morpho-agronomic and quality traits identified in durum wheat landraces.

Category	Gene/QTL Trait	Landrace (Origin)	Analysis Type	References
Biotic stress	Fusarium head blight resistance	Tunisia	GWAS	Ghavami et al., 2011
	Fusarium head blight resistance	Syria	Trait phenotyping	Talas et al., 2011
	Leaf and stem rust resistance	diverse	Gene/QTL mapping	Aoun et al., 2017
	Leaf and stem rust resistance	Portugal	Gene/QTL mapping	Aoun et al., 2019
	Leaf and stem rust resistance	Kazakhstan	GWAS	Genievskaya et al., 2022
	Leaf rust resistance	diverse	GWAS	Aoun et al., 2016
	Leaf rust resistance	Portugal	Gene/QTL mapping	Qureshi et al., 2017
	Leaf rust resistance	Portugal	Gene/QTL mapping	Qureshi et al., 2018
	Leaf rust resistance	Middle Est	Gene/QTL mapping	Kthiri et al., 2019
	Resistance to common bunt	Syria	Trait phenotyping	Mamluk and Nachit, 1994
	Septoria tritici blotch disease resistance	Tunisia	Gene/QTL mapping	Medini et al., 2014
	Septoria tritici blotch disease resistance	Tunisia	Trait phenotyping	Ferjaoui et al., 2015
	Septoria tritici blotch disease resistance	Ethiopia	GWAS	Kidane et al., 2017b
	Septoria tritici blotch disease resistance	Tunisia	Gene/QTL mapping	Aouini, 2018
	Septoria tritici blotch disease resistance	Tunisia	Trait phenotyping	Ouaja et al., 2020
	Septoria tritici blotch disease resistance	diverse	Trait phenotyping	Ben M’Barek et al., 2022
	Septoria tritici blotch disease resistance	Tunisia	Gene/QTL mapping	Ferjaoui et al., 2022
	Stem rust resistance	Italy	GWAS	Laidò et al., 2015
	Stem rust resistance	diverse	GWAS	Chao et al., 2017
	Stem rust resistance	Ethiopia	GWAS	Liu et al., 2017
	Stem rust resistance	Italy	GWAS	Saccomanno et al., 2018
	Stem rust resistance	diverse	Trait phenotyping	Olivera et al., 2021
	Stem rust resistance	Ethiopia	Trait phenotyping	Chiko et al., 2022
	Stem rust resistance	Iran	GWAS	Mehrabi et al., 2022
	Stem sawfy resistance	diverse	Gene/QTL mapping	Varella et al., 2019
	Tan spot resistance	diverse	Trait phenotyping	Laribi et al., 2021
	Yellow/stripe leaf and stem rust resistance	diverse	Association analysis/single marker scan	Bansal et al., 2013
	Yellow/stripe rust and common bunt resistance	diverse	Trait phenotyping	Annicchiarico et al., 1995
	Yellow/stripe rust resistance	Ethiopia	GWAS	Alemu et al., 2021
Abiotic stress	Allelophaty	Italy	Trait phenotyping	Scavo et al., 2022
	Cold	Iran	Trait phenotyping	Mohammadi et al., 2014
	Drought	diverse	Trait phenotyping	Pecetti et al., 1994
	Drought	diverse	Trait phenotyping	Annicchiarico et al., 1995
	Drought	Jordania	Trait phenotyping	Al Khateeb et al., 2017
	Drought	Israeli Palestina	Trait phenotyping	Frankin et al., 2021
	Drought	diverse	GWAS	Wang et al., 2019
	Heat	diverse	Trait phenotyping	Sareen et al., 2020
	Heat	Spain	Trait phenotyping	Naranjo et al., 2022
	Heat	Italy	Trait phenotyping	Taranto et al., 2022
	Salinity	Afganistan	Trait phenotyping	Shavrukov et al., 2011
	Salinity	Afganistan	QTL Mapping	Shamaya et al., 2017
	Salinity	Italy	Trait phenotyping	Maucieri et al., 2018
	Salinity	Tunisia	Gene functional characterization	Hamdi et al., 2020
	Salinity	Jordania	Trait phenotyping	Al Khateeb et al., 2020
Agronomic traits	Agromorphological traits	Marocco	Trait phenotyping	Taghouti et al., 2013
	Agromorphological traits, phenology	Italy	Trait phenotyping	Fiore et al., 2019
	Agromorphological traits (phenology, yield and morphology)	Spain	Trait phenotyping	Ruiz et al., 2012
	Agronomic (plant height, yield traits and phenology) and physiology trait	diverse	GWAS	Royo et al., 2021
	Agronomic trait (phenology, biomass and yield plant height)	Ethiopia	GWAS	Mengistu et al., 2016
	Agronomic trait (phenology, biomass and yield plant height)	diverse	Trait phenotyping	Royo et al., 2014
	Flowering time	diverse	GWAS	Gupta et al., 2020
	Flowering time, yield	diverse	GWAS	Royo et al., 2020
	Heading date, seed weight, and morphology	Iran	Gene/QTL mapping	Desiderio et al., 2019
	Morphology, phenology, yield component, GXE interaction	Ethiopia	Trait phenotyping	Mulugeta et al., 2022
	Morphology and yield	diverse	GWAS	Wang et al., 2019
	Morphology and yield, descriptors pigmentation, phenology	Oman	Trait phenotyping	Al Lawati et al., 2021
	Phenology, plant height, yield, and yield components	Ethiopia	GWAS	Kidane et al., 2017a
	Root system architecture traits	Ethiopia	GWAS	Alemu et al., 2021
	Spike height and shape	Marocco	Trait phenotyping	Sahri et al., 2014
	Stem cross section height and heading date	Spain	GWAS	Ávila et al., 2021
	Yield component	India	GWAS	Sukumaran et al., 2018
	Yield component	diverse	Trait phenotyping	Pecetti et al., 1994
	Yield component (kernel and spikes) and heading date	Italy	Trait phenotyping	Marzario et al., 2018
	Yield component, plant height, phenology and biomass	diverse	GWAS	Soriano et al., 2018
	Yield phenology lodging resistance	Israeli Palestina	Trait phenotyping	Frankin et al., 2021
	Yield vigour, plant height, phenology	diverse	Trait phenotyping	Annicchiarico et al., 1995
	Yield vigour, plant height, phenology	Algeria	Trait phenotyping	Annicchiarico et al., 2009
Quality	Arabinoxylan iron zinc phytate and phenolic acids content	Iran Mexico	Trait phenotyping	Hernandez-Espinosa et al., 2020
	Carotenoid content	Spain	Trait phenotyping	Requena-Ramírez et al., 2021
	Carotenoid content	Spain	GWAS	Requena-Ramírez et al., 2022
	Carotenoid content, color characteristics, chemical composition and starch digestibility	Italy	Trait phenotyping	Melini et al., 2021
	Gliadins content	Bulgaria	Trait phenotyping	Melnikova et al., 2010
	Gluten strength	Spain, CIMMYT, Italy, France and US	Trait phenotyping	Nazco et al., 2014b
	Glutenin protein composition	diverse	Trait phenotyping	Moragues et al., 2006
	Low molecular weight glutenin	Spain	Allelic variation	Ruiz et al., 2018
	High molecular weight glutenin	Italy	Mass spectrometry	Visioli et al., 2021
	Grain morphology and color	diverse	GWAS	Chou et al., 2022
	Grain quality	Marocco	Trait phenotyping	Taghouti et al., 2013
	Grain quality	Mexico	Trait phenotyping	Hernández-Espinosa et al., 2018
	Grain quality, yield, protein content, gluten strength and yellow color index	diverse	Trait phenotyping	Nazco et al., 2012
	Malting brewing related traits	Italy	Trait phenotyping	Alfeo et al., 2018
	Morpho-physiological characters	diverse	Trait phenotyping	Annicchiarico et al., 1995
	Phenolic and flavonoid content	Italy	Trait phenotyping	Dinelli et al., 2009
	Phenolic content	Italy	Trait phenotyping	Lo Bianco et al., 2017
	Physico-chemical traits, malt related traits, sugars	Italy	Trait phenotyping	Alfeo et al., 2021
	Phytochemical, antioxidant capacity and phenolic acids	Italy	Trait phenotyping	Di Loreto et al., 2018
	Polyphenolic content and antioxidants	Tunisia	Trait phenotyping	Boukid et al., 2019
	Prolamins	Spain	Trait phenotyping	Chacón et al., 2020
	Protein content, dry gluten gluten index, yellow index, ash P/L W G baking aptitude	Italy	Trait phenotyping	Ruisi et al., 2021
	Proteomic profiling (Metabolic and CM-protein fraction)	Italy	Trait phenotyping	Di Francesco et al., 2020
	Quality and rheological traits	diverse	Trait phenotyping	Ladhari et al., 2022
	Quality traits	Ethiopia	Trait phenotyping	Dagnaw et al., 2022
	Quality traits	Spain	Meta-QTL analysis	Roselló et al., 2018
	Quality traits and nitrogen use efficency	Tunisia	Trait phenotyping	Ayadi et al., 2022
	Quality traits	Italy	Trait phenotyping	Fiore et al., 2019
	Rheological parameters	Italy	Trait phenotyping	Spina et al., 2021
	Volatile organic compounds proteins	Italy	Trait phenotyping	Vita et al., 2016

Besides introducing new abiotic stresses, climate changes are shaping the dynamics of plants and pathogens resulting in more complex biological interactions difficult to predict and characterized by new outbreaks. Therefore, landraces are being widely explored as a potential source of new resistance traits, in regions where plant and pathogens co-evolved. Resistance sources to *Fusarium* head blight, rust, common bunt, stem sawfly, tan spot, and *Septoria tritici* blotch disease have been discovered within different DW landrace collections and majority of these have also been successfully mapped in the latest years. As pivotal examples leaf and stem rust resistance sources were mapped in the Portuguese DW landraces PI 192051 and Aus26582, by developing RIL mapping populations, beside alternative contribution from other sources (Qureshi et al., 2017; Qureshi et al., 2018; Aoun et al., 2019). Similarly, a resistance gene to *Zymoseptoria tritici* was mapped in the Tunisian DW landrace ‘Agili 39’ (Ferjaoui et al., 2022).

Concerning abiotic stress resistance, it is well recognized that Mediterranean DW landraces represent a particularly important group because of their documented better adaptation to drought (Moragues et al., 2006; Royo et al., 2014). Therefore, landraces from this region potentially include adaptive traits that could be exploited to boost the breeding for heat/drought tolerance and promote cultivars adaptation to stress-prone environments. Up to now, drought, heat, and salinity resistance traits have been studied in landraces coming from the Mediterranean basin, such as in Jordanian, Israeli-Palestinian, Tunisian, Italian, and Spanish, but also Afghan landraces (Al Khateeb et al., 2017; Shamaya et al., 2017; Hamdi et al., 2020; Frankin et al., 2021; Naranjo et al., 2022; Taranto et al., 2022). However, works concerning the mapping analysis of resistance traits to abiotic stresses are less in number than the biotic stresses ones. Just in two cases, abiotic stress resistance sources have been genetically mapped. A salinity resistance trait has been identified in an Afghan DW landrace and mapped. Moreover, using a GWAS approach on a worldwide collection of DW landraces, drought stress tolerance was associated to a locus of DW genome found to be collinear with a previously identified QTL in bread wheat (Shamaya et al., 2017; Wang et al., 2019). In order to improve the genetic characterization of abiotic resistance traits from landraces, an important contribution is expected from the development of adequate protocols for the abiotic stress evaluation, following similar strategies to those applied in bread wheat for high-throughput and accurate stress response phenotyping in large collections (Rasheed and Xia, 2019; Langridge et al., 2021; Shan et al., 2022).

Landraces are typically low yielding and can show lower agronomic attributes. Therefore, several studies enquired agronomic traits variability in landraces, focusing mainly on yield, phenology and morphological traits (**Table 3**). GWAS studies, based on high-throughput genotyping tools, helped in defining genomic regions affecting such agronomic traits in landraces highlighting available superior alleles. Among others, the contribution to the yield of root system architecture traits and phenology were highlighted (Mengistu et al., 2016; Kidane et al., 2017a; Desiderio et al., 2019; Gupta et al., 2020; Alemu et al., 2021; Ávila et al., 2021; Royo et al., 2021).

## Quality traits of durum wheat landraces

DW semolina is considered the ideal raw material for the production of pasta or macaroni products, especially in Italy, which is the first producer and consumer of DW in Europe (http://www.internationalpasta.org, accessed on 12 November 2022). The aptitude of the raw material to be transformed into a high quality end-product mainly depends on grain protein content (GPC) and composition that directly affect wheat’s market price and end-use value (Shewry, 2019).

Grain protein content, mainly above 12-13%, is highly related to the amount and composition of glutenins and gliadins proteins, that are the principal components of gluten and are responsible for the viscoelastic properties and extensibility of semolina, respectively. Past breeding activities aimed at improving grain yield resulted in a loss of genetic variability for quality-related traits, probably because of the negative relationship between yield and GPC (Nazco et al., 2014a; Subira et al., 2014). As proof of this, Roncallo et al. (2021) observed a decreasing trend in GPC over the last 85 years using a DW collection including accessions representative of the Argentina, Italy, Chile, France, CIMMYT and other countries breeding programs.

Previous studies suggested the potential quality-enhancing landraces as reservoir of new allelic variants for gluten quality improvement (**Table 3**) (Moragues et al., 2006; Nazco et al., 2014b; Roselló et al., 2018; Ruiz et al., 2018; Hernández-Espinosa et al., 2018). Roselló et al. (2018) performed a pasta-making quality QTLome using a Mediterranean collection of DW landraces and observed how landraces had higher GPC than modern cultivars but lower gluten strength. This result is due to very few allelic combinations of glutenin subunit loci in modern cultivars (Nazco et al., 2014b), while landraces showed a higher genetic variability useful to recovering and broadening allelic variation of gluten composition.

Other parameters can affect pasta production such as color (**Table 3**). Semolina and pasta color are constituted by yellow (desirable) and brown (undesirable) pigments (Colasuonno et al., 2019). Usually, DW landraces showed lower total carotenoid contents and higher values of browning compounds compared to commercial cultivars (Digesù et al., 2009; Subira et al., 2014; Taranto et al., 2015). However, the first DW landraces with carotenoid esterification ability were identified by Requena-Ramírez et al. (2021) and could represent donor sources in DW biofortification programs.

Although DW is mostly used for pasta production, it is an ingredient in typical breads in some areas of Southern Italy. It is the case of “Pane nero di Castelvetrano” and “Pane di Monreale” which are two traditional breads constituted by two Sicilian landraces, Timilia and Russello (Palumbo et al., 2008; Melini et al., 2021; Visioli et al., 2021). The most notable characteristic of Timilia is the dark color of semolina, due to the high content of antioxidant phenolic compounds (Giancaspro et al., 2016; Bianco et al., 2017; Taranto et al., 2022). To preserve these landraces and derived-products, a traceability approach was developed using the high molecular weight glutenins, suggesting a method to verify the varietal identity from the seed to the final product (Visioli et al., 2021).

## Conclusions

In conclusion, recent findings unveiled the strategic role of landraces in the genetic improvement of durum wheat. Studies on genomic divergence among *T. turgidum* sub-species indicated that the allelic variations of domesticated accessions and their wild relatives, lost during the domestication and breeding processes, were and will be recovered by exploring and exploiting landraces genetic diversity. In particular, in a context of climate changes, understanding the environmental and genetic factors behind the adaptation of landraces can help to introduce beneficial alleles in elite varieties to overcome stress and increase yield. The availability of durum wheat reference genome and the increasingly precise molecular techniques at affordable costs are giving a big boost to accurately identify the genetic determinants underpinning resistance/tolerance against biotic and abiotic stresses.

The recent application of genomic technologies (i.e. genome-wide association and genomic prediction analysis) on durum wheat landrace resources paves the way to accelerate the next-generation breeding programs to overcome the gap of knowledge of these underexplored resources and identify advantageous alleles that have been lost in modern varieties.

## Author contributions

CB: writing original draft. DB and FT: conceptualization and writing. FT, GD, AF: writing, review, and editing. All authors contributed to the article and approved the submitted version.

## References

[B1001] Abu-Zaitoun S. Y. Chandrasekhar K. Assili S. Shtaya M. J. Jamous R. M. Mallah O. B.. (2018). Unlocking the genetic diversity within a middle-East panel of durum wheat landraces for adaptation to semi-arid climate 233. doi: 10.3390/agronomy8100233

[B1] Adhikari S. Kumari J. Jacob S. R. Prasad P. Gangwar O. P. Lata C.. (2022). Landraces-potential treasure for sustainable wheat improvement. Genet. Resour. Crop Evol. 69, 499–523. doi: 10.1007/s10722-021-01310-5

[B2] Alahmad S. Dinglasan E. Leung K. M. Riaz A. Derbal N. Voss-Fels K. P.. (2018). Speed breeding for multiple quantitative traits in durum wheat. Plant Methods 14, 1–15. doi: 10.1186/s13007-018-0302-y 29785201 PMC5950182

[B3] Alemu Y. A. Anley A. M. Abebe T. D. (2020). Genetic variability and association of traits in Ethiopian durum wheat (*Triticum turgidium* l. var. *durum*) landraces at dabat research station, north gondar. Cogent. Food Agric. 6, 1778604. doi: 10.1080/23311932.2020.1778604

[B4] Alemu S. K. Huluka A. B. Tesfaye K. Haileselassie T. Uauy C. (2021). Genome-wide association mapping identifies yellow rust resistance loci in Ethiopian durum wheat germplasm. PloS One 16 (5), e0243675. doi: 10.1371/journal.pone.0243675 33999918 PMC8128278

[B6] Alfeo V. De Francesco G. Sileoni V. Blangiforti S. Palmeri R. Aerts G.. (2021). Physicochemical properties, sugar profile, and non-starch polysaccharides characterization of old wheat malt landraces. J. Food Compos. Anal. 102, 103997. doi: 10.1016/j.jfca.2021.103997

[B5] Alfeo V. Jaskula-Goiris B. Venora G. Schimmenti E. Aerts G. Todaro A. (2018). Screening of durum wheat landraces (*Triticum turgidum* subsp. *durum*) for the malting suitability. J. Cereal Sci. 83, 10. doi: 10.1016/j.jcs.2018.08.001

[B7] Alipour H. Bihamta M. R. Mohammadi V. Peyghambari S. A. Bai G. Zhang G. (2017). Genotyping-by-sequencing (GBS) revealed molecular genetic diversity of Iranian wheat landraces and cultivars. Front. Plant Sci. 8, 1293. doi: 10.3389/fpls.2017.01293 28912785 PMC5583605

[B8] Al Khateeb W. Al Shalabi A. Schroeder D. Musallam I. (2017). Phenotypic and molecular variation in drought tolerance of Jordanian durum wheat (*Triticum durum* desf.) landraces. Physiol. Mol. Biol. 23, 311–319. doi: 10.1007/s12298-017-0434-y PMC5391366 28461720

[B9] Al Lawati A. H. Nadaf S. K. AlSaady N. A. Al Hinai S. A. Almamari A. Al Adawi M. H.. (2021). Genetic diversity of omani durum wheat (sub sp.) landraces. Open Agric. 15, 21–32. doi: 10.2174/1874331502115010021

[B10] Allen A. M. Winfield M. O. Burridge A. J. Downie R. C. Benbow H. R. Barker G. L.. (2017). Characterization of a wheat breeders’ array suitable for high-throughput SNP genotyping of global accessions of hexaploid bread wheat (*Triticum aestivum*). Plant Biotechnol. J. 15 (3), 390–401. doi: 10.1111/pbi.12635 27627182 PMC5316916

[B12] Alsaleh A. Bektas H. Baloch F. S. Nadeem M. A. Özkan H. (2022). Turkish Durum wheat conserved ex-situ and *in situ* unveils a new hotspot of unexplored genetic diversity. Crop Sci. 62, 1200–1212. doi: 10.1002/csc2.20723

[B11] Altamore L. Ingrassia M. Columba P. Chironi S. Bacarella S. (2020). Italian Consumers’ preferences for pasta and consumption trends: Tradition or innovation? J. Int. Food Agribus. 32, 337–360. doi: 10.1080/08974438.2019.1650865

[B14] Annicchiarico P. Abdellaoui Z. Kelkouli M. Zerargui H. (2005). Grain yield, straw yield and economic value of tall and semi-dwarf durum wheat cultivars in Algeria. J. Agric. Sci. 143, 57–64. doi: 10.1017/S0021859605004855

[B13] Annicchiarico P. Pecetti L. Damania A. B. (1995). Relationships between phenotypic variation and climatic factors at collecting sites in durum wheat landraces. Hereditas 122, 163–167. doi: 10.1111/j.1601-5223.1995.00163.x

[B15] Annicchiarico P. Royo C. Bellah F. Moragues M. (2009). Relationships among adaptation patterns, morphophysiological traits and molecular markers in durum wheat. Plant Breed. 128, 164–171. doi: 10.1111/j.1439-0523.2008.01557.x

[B16] Annunziata M. G. Ciarmiello L. F. Woodrow P. Maximova E. Fuggi A. Carillo P. (2017). Durum wheat roots adapt to salinity remodeling the cellular content of nitrogen metabolites and sucrose. Front. Plant Sci. 7, 2035. doi: 10.3389/fpls.2016.02035 28119716 PMC5220018

[B17] Aouini L. (2018). Durum wheat and septoria tritici blotch: genes and prospects for breeding (Order No. 28232876) Available from ProQuest Dissertations & Theses Global. (2564078719). Retrieved from https://www.proquest.com/dissertations-theses/durum-wheat-septoria-tritici-blotch-genes/docview/2564078719/se-2.

[B18] Aoun M. Breiland M. Kathryn Turner M. Loladze A. Chao S. Xu S. S.. (2016). Genome-wide association mapping of leaf rust response in a durum wheat worldwide germplasm collection. Plant Genome 9 (3). doi: 10.3835/plantgenome2016.01.0008 27902791

[B19] Aoun M. Kolmer J. A. Rouse M. N. Chao S. Bulbula W. D. Elias E. M.. (2017). Inheritance and bulked segregant analysis of leaf rust and stem rust resistance in durum wheat genotypes. Phytopathology 107, 1496–1506. doi: 10.1094/PHYTO-12-16-0444-R 28745102 PMC7779972

[B20] Aoun M. Kolmer J. A. Rouse M. N. Elias E. M. Breiland M. Bulbula W. D.. (2019). Mapping of novel leaf rust and stem rust resistance genes in the Portuguese durum wheat landrace PI 192051. G3: Genes Genomes Genet. 9, 2535–2547. doi: 10.1534/g3.119.400292 PMC6686931 31278174

[B21] Ávila C. M. Requena-Ramírez M. D. Rodríguez-Suárez C. Flores F. Sillero J. C. Atienza S. G. (2021). Genome-wide association analysis for stem cross section properties, height and heading date in a collection of spanish durum wheat landraces. Plants 10, 1123. doi: 10.3390/plants10061123 34205906 PMC8230085

[B22] Ayadi S. Jallouli S. Chamekh Z. Zouari I. Landi S. Hammami Z.. (2022). Variation of grain yield, grain protein content and nitrogen use efficiency components under different nitrogen rates in mediterranean durum wheat genotypes. Agriculture 12, 916. doi: 10.3390/agriculture12070916

[B23] Ayalew H. Tsang P. W. Chu C. Wang J. Liu S. Chen C.. (2019). Comparison of TaqMan, KASP and rhAmp SNP genotyping platforms in hexaploid wheat. PloS One 14 (5), e0217222. doi: 10.1371/journal.pone.0217222 31116793 PMC6530864

[B24] Azeez A. M. Adubi O. A. Durodola F. A. (2018). “Landraces and crop genetic improvement,” in Rediscovery of landraces as a resource for the future, IntechOpen, London, UK, 1–19.

[B29] Balla M. Y. Gorafi Y. S. A. Kamal N. M. Abdalla M. G. A. Tahir I. S. A. Tsujimoto H. (2022). Harnessing the diversity of wild emmer wheat for genetic improvement of durum wheat. Theor. Appl. Genet. 135, 1671–1684. doi: 10.1007/s00122-022-04062-7 35257197 PMC9110450

[B30] Bansal U. K. Arief V. N. DeLacy I. H. Bariana H. S. (2013). Exploring wheat landraces for rust resistance using a single marker scan. Euphytica 194, 219–233. doi: 10.1007/s10681-013-0940-0

[B27] Bayer P. E. Golicz A. A. Scheben A. Batley J. Edwards D. (2020). Plant pan-genomes are the new reference. Nat. Plants 6, 914–920. doi: 10.1038/s41477-020-0733-0 32690893

[B28] Bayer P. E. Petereit J. Durant E. Monat C. Rouard M. Hu H.. (2022). Bread wheat genomes graph pangenome. Zenodo. doi: 10.1101/2022.02.23.481560 PMC12806990 35644986

[B31] Ben M’Barek S. Laribi M. Kouki H. Castillo D. Araar C. Nefzaoui M.. (2022). Phenotyping Mediterranean durum wheat landraces for resistance to *Zymoseptoria tritici* in Tunisia. Genes 13, 355. doi: 10.3390/genes13020355 35205399 PMC8872163

[B32] Bianco M. L. Siracusa L. Dattilo S. Venora G. Ruberto G. (2017). Phenolic fingerprint of sicilian modern cultivars and durum wheat landraces: a tool to assess biodiversity. Cereal Chem. 94, 1045–1051. doi: 10.1094/CCHEM-06-17-0125-R

[B33] Borrill P. Harrington S. A. Uauy C. (2019). Applying the latest advances in genomics and phenomics for trait discovery in polyploid wheat. Plant J. 97, 56–72. doi: 10.1111/tpj.14150 30407665 PMC6378701

[B34] Boukid F. Dall’Asta M. Bresciani L. Mena P. Del Rio D. Calani L.. (2019). Phenolic profile and antioxidant capacity of landraces, old and modern Tunisian durum wheat. Eur. Food Res. Technol. 245, 73–82. doi: 10.1007/s00217-018-3141-1

[B35] Bradbury P. J. Zhang Z. Kroon D. E. Casstevens T. M. Ramdoss Y. Buckler E. S. (2007). TASSEL: Software for association mapping of complex traits in diverse samples. Bioinformatics 23, 2633–2635. doi: 10.1093/bioinformatics/btm308 17586829

[B36] Broccanello C. Chiodi C. Funk A. McGrath J. M. Panella L. Stevanato P. (2018). Comparison of three PCR-based assays for SNP genotyping in plants. Plant Methods 14, 1–8. doi: 10.1186/s13007-018-0295-6 29610576 PMC5872507

[B37] Broccanello C. Gerace L. Stevanato P. (2020). “QuantStudio™ 12K flex OpenArray® system as a tool for high-throughput genotyping and gene expression analysis,” in Quantitative real-time PCR (New York, NY: Humana), 199–208. 10.1007/978-1-4939-9833-3_15 31578697

[B38] Camacho Villa T. C. Maxted N. Scholten M. Ford-Lloyd B. (2005). Defining and identifying crop landraces. Plant Genet. Resour. Charact. Util. 3, 373–384. doi: 10.1079/PGR200591

[B40] Capblancq T. Forester B. R. (2021). Redundancy analysis: A Swiss army knife for landscape genomics. Methods Ecol. Evol. 12, 2298–2309. doi: 10.1111/2041-210X.13722

[B41] Casañas F. Simó J. Casals J. Prohens J. (2017). Toward an evolved concept of landrace. Front. Plant Sci. 8, 145. doi: 10.3389/fpls.2017.00145 28228769 PMC5296298

[B1002] Capblancq T. Fitzpatrick M. C. Bay R. A. Exposito-Alonso M. Keller S. R. (2020). Genomic prediction of (mal) adaptation across current and future climatic landscapes. Annu. Rev. Ecology Evolution Systematics 51 (1). doi: 10.1146/annurev-ecolsys-020720-042553

[B1003] Cavanagh C. R. Chao S. Wang S. Huang B. E. Stephen S. Kiani S.. (2013). Genome-wide comparative diversity uncovers multiple targets of selection for improvement in hexaploid wheat landraces and cultivars. Proc. Natl. Acad. Sci. U.S.A. 110, 8057–8062. doi: 10.1073/pnas.1217133110 23630259 PMC3657823

[B39] Cavanagh C. R. Chao S. Wang S. Huang B. E. Stephen S. Kiani S.. (2020). Genomic prediction of (Mal)adaptation across current and future climatic landscapes. Annu. Rev. Ecol. Evol. 51, 245–271. doi: 10.1146/annurev-ecolsys-020720-042553

[B42] Chacón E. A. Vázquez F. J. Giraldo P. Carrillo J. M. Benavente E. Rodríguez-Quijano M. (2020). Allelic variation for prolamins in Spanish durum wheat landraces and its relationship with quality traits. Agronomy 10, .136. doi: 10.3390/agronomy10010136

[B1004] Chao S. Rouse M. N. Acevedo M. Szabo-Hever A. Bockelman H. Bonman J. M.. (2017). Evaluation of genetic diversity and host resistance to stem rust in USDA NSGC durum wheat accessions. Plant Genome. 10 (2). doi: 10.3835/plantgenome2016.07.0071 28724063

[B43] Chaparro-Encinas L. A. Santoyo G. Peña-Cabriales J. J. Castro-Espinoza L. Parra-Cota F. I. Santos-Villalobos S. D. L. (2021). Transcriptional regulation of metabolic and cellular processes in durum wheat (*Triticum turgidum* subsp. *durum*) in the face of temperature increasing. Plants 10, 2792. doi: 10.3390/plants10122792 34961263 PMC8703274

[B44] Chaurasia S. Singh A. K. Songachan L. S. Sharma A. D. Bhardwaj R. Singh K. (2020). Multi-locus genome-wide association studies reveal novel genomic regions associated with vegetative stage salt tolerance in bread wheat (*Triticum aestivum* l.). Genomics 112, 4608–4621. doi: 10.1016/j.ygeno.2020.08.006 32771624

[B45] Chiko S. Kebede Gessese M. Shimelash D. Haile W. T. Melo B. Y. Wassie A. S.. (2022). Diversity of Ethiopian durum wheat landraces for resistance to stem rust seedling resistance renes. Adv. Agric. 2022. doi: 10.1155/2022/3023427

[B1005] Chou C. H. Lin H. S. Wen C. H. Tung C. W. (2022). Patterns of genetic variation and QTLs controlling grain traits in a collection of global wheat germplasm revealed by high-quality SNP markers. BMC Plant Biol. 22, 455. doi: 10.1186/s12870-022-03844-x 36131260 PMC9494784

[B46] Chung Y. S. Choi S. C. Jun T. H. Kim C. (2017). Genotyping-by-sequencing: a promising tool for plant genetics research and breeding. Horticult. Environ. Biotechnol. 58, 425–431. doi: 10.1007/s13580-017-0297-8

[B48] Colasuonno P. Marcotuli I. Blanco A. Maccaferri M. Condorelli G. E. Tuberosa R.. (2019). Carotenoid pigment content in durum wheat (*Triticum turgidum l.* var *durum*): An overview of quantitative trait loci and candidate genes. Front. Plant Sci. 10, 1347. doi: 10.3389/fpls.2019.01347 31787991 PMC6853866

[B47] Colasuonno P. Marcotuli I. Gadaleta A. Soriano J. M. (2021). From genetic maps to QTL cloning: an overview for durum wheat. Plants 10, 315. doi: 10.3390/plants10020315 33562160 PMC7914919

[B49] Colmer T. D. Munns R. Flowers T. J. (2005). Improving salt tolerance of wheat and barley: future prospects. Aust. J. Exp. Agric. 45, 1425–1443. doi: 10.1071/EA04162

[B50] Crossa J. Jarquín D. Franco J. Pérez-Rodríguez P. Burgueño J. Saint-Pierre C.. (2016). Genomic prediction of gene bank wheat landraces. G3: Genes Genomes Genet. 6, 1819–1834. doi: 10.1534/g3.116.029637 PMC4938637 27172218

[B51] Crossa J. Pérez-Rodríguez P. Cuevas J. Montesinos-López O. Jarquín D. De Los Campos G.. (2017). Genomic selection in plant breeding: methods, models, and perspectives. Trends Plant Sci. 22, 961–975. doi: 10.1016/j.tplants.2017.08.011 28965742

[B52] Daetwyler H. D. Pong-Wong R. Villanueva B. Woolliams J. A. (2010). The impact of genetic architecture on genome-wide evaluation methods. Genetics 185, 1021–1031. doi: 10.1534/genetics.110.116855 20407128 PMC2907189

[B53] Dagnaw T. Mulugeta B. Haileselassie T. Geleta M. Tesfaye K. (2022). Phenotypic variability, heritability and associations of agronomic and quality traits in cultivated Ethiopian durum wheat (*Triticum turgidum* l. ssp. *durum*, desf.). Agronomy 12, 1714. doi: 10.3390/agronomy12071714

[B54] Davenport R. James R. A. Zakrisson-Plogander A. Tester M. Munns R. (2005). Control of sodium transport in durum wheat. Plant Physiol. 137, 807–818. doi: 10.1104/pp.104.057307 15734907 PMC1065380

[B55] Davey J. W. Hohenlohe P. A. Etter P. D. Boone J. Q. Catchen J. M. Blaxter M. L. (2011). Genome-wide genetic marker discovery and genotyping using next-generation sequencing. Nat. Rev. Genet. 12, 499–510. doi: 10.1038/nrg3012 21681211

[B56] De Cillis U. (1942). I frumenti Siciliani. Stazione Sperimentale di Granicoltura "Benito Mussolini" per la Sicilia - Catania. Pubblicazione n. 9 p. 1–323.

[B1006] Desiderio F. Zarei L. Licciardello S. Cheghamirza K. Farshadfar E. Virzi N.. (2019). Genomic regions from an Iranian landrace increase kernel size in durum wheat. Front. Plant Sci. 10. doi: 10.3389/fpls.2019.00448 PMC6482228 31057571

[B57] De Santis M. A. Soccio M. Laus M. N. Flagella Z. (2021). Influence of drought and salt stress on durum wheat grain quality and composition: A review. Plants 10, 2599. doi: 10.3390/plants10122599 34961071 PMC8708103

[B58] Deschamps S. Llaca V. May G. D. (2012). Genotyping-by-sequencing in plants. Biology 1, 460–483. doi: 10.3390/biology1030460 24832503 PMC4009820

[B59] De Vita P. Taranto F. (2019). “Durum wheat (Triticum turgidum ssp. durum) breeding to meet the challenge of climate change,” in Advances in plant breeding strategies: cereals (Switzerland: Springer, Cham).

[B60] Di Francesco A. Saletti R. Cunsolo V. Svensson B. Muccilli V. De Vita P.. (2020). Qualitative proteomic comparison of metabolic and CM-like protein fractions in old and modern wheat Italian genotypes by a shotgun approach. J. Proteomics. 211, 103530. doi: 10.1016/j.jprot.2019.103530 31629055

[B61] Digesù A. M. Platani C. Cattivelli L. Mangini G. Blanco A. (2009). Genetic variability in yellow pigment components in cultivated and wild tetraploid wheats. J. Cereal Sci. 50 (2), 210–218. doi: 10.1016/j.jcs.2009.05.002

[B62] Di Loreto A. Bosi S. Montero L. Bregola V. Marotti I. Sferrazza R. E.. (2018). Determination of phenolic compounds in ancient and modern durum wheat genotypes. Electrophoresis 39, 2001–2010. doi: 10.1002/elps.201700439 29569730

[B63] Dinelli G. Carretero A. S. Di Silvestro R. Marotti I. Fu S. Benedettelli S.. (2009). Determination of phenolic compounds in modern and old varieties of durum wheat using liquid chromatography coupled with time-of-flight mass spectrometry. J. Chromatogr. A. 1216, 7229–7240. doi: 10.1016/j.chroma.2009.08.041 19740468

[B64] Di Pede G. Dodi R. Scarpa C. Brighenti F. Dall’Asta M. Scazzina F. (2021). Glycemic index values of pasta products: An overview. Foods 10, 2541. doi: 10.3390/foods10112541 34828822 PMC8623826

[B65] Dvorak P. Krasylenko Y. Zeiner A. Šamaj J. Takác T. (2021). Signaling toward reactive oxygen species-scavenging enzymes in plants. Front. Plant Sci. 11, 618835. doi: 10.3389/fpls.2020.618835 33597960 PMC7882706

[B66] Edelstein M. Plaut Z. Ben-Hur M. (2010). Water salinity and sodicity effects on soil structure and hydraulic properties. Adv. Hortic. Sci. 24, 154–160.

[B67] Elshire R. J. Glaubitz J. C. Sun Q. Poland J. A. Kawamoto K. Buckler E. S.. (2011). A robust, simple genotyping-by-sequencing (GBS) approach for high diversity species. PloS One 6, 19379. doi: 10.1371/journal.pone.0019379 PMC3087801 21573248

[B68] Esposito S. Taranto F. Vitale P. Ficco D. B. M. Colecchia S. A. Stevanato P.. (2022). Unlocking the molecular basis of wheat straw composition and morphological traits through multi-locus GWAS. BMC Plant Biol. 22, 1–19. doi: 10.1186/s12870-022-03900-6 36344939 PMC9641881

[B1000] Etminan A. Pour-Aboughadareh A. Mohammadi R. Ahmadi-Rad A. Moradi Z. Mahdavian Z.. (2017). Evaluation of genetic diversity in a mini core collection of Iranian durum wheat germplasms. J. Anim. Plant Sci. 27, 1582–1587.

[B69] Fayaz F. Aghaee Sarbarzeh M. Talebi R. Azadi A. (2019). Genetic diversity and molecular characterization of Iranian durum wheat landraces (*Triticum turgidum durum* (Desf.) husn.) using DArT markers. Biochem. Genet. 57, 98–116. doi: 10.1007/s10528-018-9877-2 30051349

[B72] Ferjaoui S. Aouini L. Slimane R. B. Ammar K. Dreisigacker S. Schouten H. J.. (2022). Deciphering resistance to *Zymoseptoria tritici* in the Tunisian durum wheat landrace accession ‘Agili39’. BMC Genom. 23, 1–20. doi: 10.1186/s12864-022-08560-2 PMC9112612 35581550

[B71] Ferjaoui S. M'Barek S. B. Bahri B. Slimane R. B. Hamza S. (2015). Identification of resistance sources to septoria tritici blotch in old Tunisian durum wheat germplasm applied for the analysis of the *Zymoseptoria tritici*-durum wheat interaction. J. Plant Pathol. 97, 1–11. doi: 10.4454/JPP.V97I3.028

[B70] Fernando R. L. Garrick D. (2013). “Bayesian Methods applied to GWAS,” in Genome wide association studies and genomic prediction, vol. 1019 . Eds. Gondro C. van der Werf J. Hayes B. (Totowa, NJ: Humana Press), 237–274. 10.1007/978-1-62703-447-0_10 23756894

[B1008] Fiore M. C. Mercati F. Spina A. Blangiforti S. Venora G. Dell'Acqua M.. (2019). High-throughput genotype, morphology, and quality traits evaluation for the assessment of genetic diversity of wheat landraces from Sicily. Plants 8, 116. doi: 10.3390/plants8050116 31052327 PMC6572038

[B1009] Flagella Z. Giuliani M. M. Giuzio L. Volpi C. Masci S. (2010). Influence of water deficit on durum wheat storage protein composition and technological quality. Eur. J. Agron. 33, 197–207. doi: 10.1016/j.eja.2010.05.006

[B74] Forester B. R. Lasky J. R. Wagner H. H. Urban D. L. (2018). Comparing methods for detecting multilocus adaptation with multivariate genotype–environment associations. Mol. Ecol. 27, 2215–2233. doi: 10.1111/mec.14584 29633402

[B73] Forrest K. Pujol V. Bulli P. Pumphrey M. Wellings C. Herrera-Foessel S.. (2014). Development of a SNP marker assay for the *Lr67* gene of wheat using a genotyping by sequencing approach. Mol. Breed. 34, 2109–2118. doi: 10.1007/s11032-014-0166-4

[B75] Frankin S. Roychowdhury R. Nashef K. Abbo S. Bonfil D. J. Ben-David R. (2021). In-field comparative study of landraces vs. modern wheat genotypes under a mediterranean climate. Plants 10, 2612. doi: 10.3390/plants10122612 34961083 PMC8705803

[B76] Ganal M. W. Polley A. Graner E.-M. Plieske J. Wieseke R. Luerssen H.. (2012). Large SNP arrays for genotyping in crop plants. J. Biosci. 37, 821–828. doi: 10.1007/s12038-012-9225-3 23107918

[B1010] Genievskaya Y. Pecchioni N. Laidò G. Anuarbek S. Rsaliyev A. Chudinov V.. (2022). Genome-wide association study of leaf rust and stem rust seedling and adult resistances in tetraploid wheat accessions harvested in kazakhstan. Plants 11, 1904. doi: 10.3390/plants11151904 35893608 PMC9329756

[B77] Ghavami F. Elias E. M. Mamidi S. Ansari O. Sargolzaei M. Adhikari T.. (2011). Mixed model association mapping for fusarium head blight resistance in Tunisian-derived durum wheat populations. G3: Genes| Genomes| Genet. 1, 209–218. doi: 10.1534/g3.111.000489 PMC3276138 22384332

[B78] Giancaspro A. Colasuonno P. Zito D. Blanco A. Pasqualone A. Gadaleta A. (2016). Varietal traceability of bread ‘Pane Nero di castelvetrano’ by denaturing high pressure liquid chromatography analysis of single nucleotide polymorphisms. Food Control 59, 809–817. doi: 10.1016/j.foodcont.2015.07.006

[B79] Golicz A. A. Bayer P. E. Barker G. C. Edger PP Kim H Martinez PA. (2016). The pangenome of an agronomically important crop plant brassica oleracea. Nat. Commun. 7, 13390. doi: 10.1038/ncomms13390 27834372 PMC5114598

[B80] Guerrero J. Andrello M. Burgarella C. Manel S. (2018). Soil environment is a key driver of adaptation in *Medicago truncatula*: new insights from landscape genomics. New Phytol. 219, 378–390. doi: 10.1111/nph.15171 29696659

[B1011] Gupta P. K. Balyan H. S. Sharma S. Kumar R. (2020). Genetics of yield, abiotic stress tolerance and biofortification in wheat (*Triticum aestivum* l.). Theor. Appl. Genet. 133, 1569–1602. doi: 10.1007/s00122-020-03583-3 32253477

[B81] Guzman C. Peña R. J. Singh R. Autrique E. Dreisigacker S. Crossa J.. (2016). Wheat quality improvement at CIMMYT and the use of genomic selection on it. Appl. Trans. Genomics 11, 3–8. doi: 10.1016/j.atg.2016.10.004 PMC5167370 28018844

[B1012] Halder J. Zhang J. Ali S. Sidhu J. S. Gill H. S. Talukder S. K.. (2019). Mining and genomic characterization of resistance to tan spot, stagonospora nodorum blotch (SNB), and fusarium head blight in Watkins core collection of wheat landraces. BMC Plant Biol. 19, 480. doi: 10.1186/s12870-019-2093-3 31703626 PMC6839225

[B1013] Hamdi K. Brini F. Kharrat N. Masmoudi K. Yakoubi I. (2020). Abscisic acid, stress, and ripening (*Tt*ASR1) gene as a functional marker for salt tolerance in durum wheat. BioMed. Res. Int. 31, 7876357. doi: 10.1155/2020/7876357 PMC7013306 32076614

[B82] Hanif U. Alipour H. Gul A. Jing L. Darvishzadeh R. Amir R.. (2021). Characterization of the genetic basis of local adaptation of wheat landraces from Iran and Pakistan using genome-wide association study. TPG 14, 20096. doi: 10.1002/tpg2.20096 PMC12807341 34275212

[B83] Harrington S. A. Cobo N. Karafiatova M. Dolezel J. Borrill P. Uauy C. (2019). Identification of a dominant chlorosis phenotype through a forward screen of the *Triticum turgidum* cv. kronos TILLING population. Front. Plant Sci. 10, 963. doi: 10.3389/fpls.2019.00963 31396255 PMC6667664

[B84] He F. Pasam R. Shi F. Kant S. Keeble-Gagnere G. Kay P.. (2019). Exome sequencing highlights the role of wild-relative introgression in shaping the adaptive landscape of the wheat genome. Nat. Genet. 5, 896–904. doi: 10.1038/s41588-019-0382-2 31043759

[B86] Hernandez-Espinosa N. Laddomada B. Payne T. Huerta-Espino J. Govindan V. Ammar K.. (2020). Nutritional quality characterization of a set of durum wheat landraces from Iran and Mexico. LWT 124, 109198. doi: 10.1016/j.lwt.2020.109198

[B85] Hernández-Espinosa N. Mondal S. Autrique E. Gonzalez-Santoyo H. Crossa J. Huerta-Espino J.. (2018). Milling, processing and end-use quality traits of CIMMYT spring bread wheat germplasm under drought and heat stress. Field Crops Res. 215, 104–112. doi: 10.1016/j.fcr.2017.10.003

[B87] Hufford M. B. Xu X. Van Heerwaarden J. Pyhäjärvi T. Chia J. M. Cartwright R. A.. (2012). Comparative population genomics of maize domestication and improvement. Nat. Genet. 44 (7), 808–811. doi: 10.1038/ng.2309 22660546 PMC5531767

[B1014] Jarquín D. Crossa J. Lacaze X. Du Cheyron P. Daucourt J. Lorgeou J.. (2014). A reaction norm model for genomic selection using high-dimensional genomic and environmental data. Theor. Appl. Genet. 127, 595–607. doi: 10.1007/s00122-013-2243-1 24337101 PMC3931944

[B88] Jin H. Wen W. Liu J. Zhai S. Zhang Y. Yan J.. (2016). Genome-wide QTL mapping for wheat processing quality parameters in a gaocheng 8901/Zhoumai 16 recombinant inbred line population. Front. Plant Sci. 7, 1032. doi: 10.3389/fpls.2016.01032 27486464 PMC4949415

[B89] Kabbaj H. Sall A. T. Al-Abdallat A. Geleta M. Amri A. Filali-Maltouf A.. (2017). Genetic diversity within a global panel of durum wheat (*Triticum durum*) landraces and modern germplasm reveals the history of allele exchange. Front. Plant Sci. 8, 1277. doi: 10.3389/fpls.2017.01277 28769970 PMC5513985

[B90] Kaur P. Gaikwad K. (2017). From genomes to GENE-omes: exome sequencing concept and applications in crop improvement. Front. Plant Sci. 8, 2164. doi: 10.3389/fpls.2017.02164 29312405 PMC5742236

[B91] Kawecki T. J. Ebert D. (2004). Conceptual issues in local adaptation. Ecol. Lett. 7, 1225–1241. doi: 10.1111/j.1461-0248.2004.00684.x

[B94] Khan A. W. Garg V. Roorkiwal M. Golicz A. A. Edwards D. Varshney R. K. (2020). Super-pangenome by integrating the wild side of a species for accelerated crop improvement. Trends Plant Sci. 25 (2), 148–158. doi: 10.1016/j.tplants.2019.10.012 31787539 PMC6988109

[B93] Kidane Y. G. Mancini C. Mengistu D. K. Frascaroli E. Fadda C. Pè M. E.. (2017a). Genome wide association study to identify the genetic base of smallholder farmer preferences of durum wheat traits. Front. Plant Sci., 1230. doi: 10.3389/fpls.2017.01230 28769945 PMC5511852

[B92] Kidane Y. G. Hailemariam B. N. Mengistu D. K. Fadda C. Pè M. E. Dell'Acqua M. (2017b). Genome-wide association study of *Septoria tritici* blotch resistance in Ethiopian durum wheat landraces. Front. Plant Sci. 8, 1586. doi: 10.3389/fpls.2017.01586 28959268 PMC5603693

[B95] Kthiri D. Loladze A. N’Diaye A. Nilsen K. T. Walkowiak S. Dreisigacker S.. (2019). Mapping of genetic loci conferring resistance to leaf rust from three globally resistant durum wheat sources. Front. Plant Sci. 10, 1247. doi: 10.3389/fpls.2019.01247 31649708 PMC6792298

[B96] Kulkarni M. Soolanayakanahally R. Ogawa S. Uga Y. Selvaraj M. G. Kagale S. (2017). Drought response in wheat: Key genes and regulatory mechanisms controlling root system architecture and transpiration efficiency. Front. Chem. 5, 106. doi: 10.3389/fchem.2017.00106 29259968 PMC5723305

[B97] Ladhari A. Corrado G. Rouphael Y. Carella F. Nappo G. R. Di Marino C.. (2022). Chemical, functional, and technological features of grains, brans, and semolina from purple and red durum wheat landraces. Foods 11, 1545. doi: 10.3390/foods11111545 35681296 PMC9180146

[B98] Laidò G. Mangini G. Taranto F. Gadaleta A. Blanco A. Cattivelli L.. (2013). Genetic diversity and population structure of tetraploid wheats (*Triticum turgidum* l.) estimated by SSR, DArT and pedigree data. PloS One 8 (6), e67280. doi: 10.1371/journal.pone.0067280 23826256 PMC3694930

[B1015] Laidò G. Panio G. Marone D. Russo M. A. Ficco D. B. Giovanniello V.. (2015). Identification of new resistance loci to African stem rust race TTKSK in tetraploid wheats based on linkage and genome-wide association mapping. Front. Plant Sci. 6. doi: 10.3389/fpls.2015.01033 PMC4673868 26697025

[B1016] Langridge P. Reynolds M. (2021). Breeding for drought and heat tolerance in wheat. Theor. Appl. Genet. 134, 1753–1769. doi: 10.1007/s00122-021-03795-1 33715017

[B99] Laribi M. Ben M’Barek S. Fakhfakh M. Yahyaoui A. H. Sassi K. (2021). Durum wheat mediterranean landraces: a valuable source for resistance to tan spot disease. Agriculture 11, 1148. doi: 10.3390/agriculture11111148

[B100] Laus M. N. De Santis M. A. Flagella Z. Soccio M. (2022). Changes in antioxidant defence system in durum wheat under hyperosmotic stress: A concise overview. Plants 11, 98. doi: 10.3390/plants11010098 PMC8747421 35009101

[B101] Lemerle D. Gill G. S. Murphy C. E. Walker S. R. Cousens R. D. Mokhtari S.. (2001). Genetic improvement and agronomy for enhanced wheat competitiveness with weeds. Aust. J. Agric. Res. 52, 527–548. doi: 10.1071/AR00056

[B1017] Lindsay M. P. Lagudah E. S. Hare R. A. Munns R. (2004). A locus for sodium exclusion (*Nax1*), a trait for salt tolerance, mapped in durum wheat. Funct. Plant Biol. 31, 1105–1114. doi: 10.1071/FP04111 32688978

[B103] Lipka A. E. Tian F. Wang Q. Peiffer J. Li M. Bradbury P. J.. (2012). GAPIT: genome association and prediction integrated tool. Bioinformatics 28, 2397–2399. doi: 10.1093/bioinformatics/bts444 22796960

[B104] Liu W. Maccaferri M. Rynearson S. Letta T. Zegeye H. Tuberosa R.. (2017). Novel sources of stripe rust resistance identified by genome-wide association mapping in Ethiopian durum wheat (*Triticum turgidum* ssp. *durum*). Front. Plant Sci. 8, 774. doi: 10.3389/fpls.2017.00774 28553306 PMC5427679

[B1018] Liu B. Asseng S. Müller C. Ewert F. Elliott J. Lobell D. B.. (2016). Similar estimates of temperature impacts on global wheat yield by three independent methods. Nat. Clim. Change. 6, 1130–1136. doi: 10.1038/nclimate3115

[B102] Li Y. F. Wu Y. Hernandez-Espinosa N. Peña R. J. (2013). Heat and drought stress on durum wheat: Responses of genotypes, yield, and quality parameters. J. Cereal Sci. 57, 398–404. doi: 10.1016/j.jcs.2013.01.005

[B105] Lopes M. S. El-Basyoni I. Baenziger P. S. Singh S. Royo C. Ozbek K.. (2015). Exploiting genetic diversity from landraces in wheat breeding for adaptation to climate change. JXB 66, 3477–3486.ù. doi: 10.1093/jxb/erv122 25821073

[B106] Maccaferri M. Harris N. S. Twardziok S. O. Pasam R. K. Gundlach H. Spannagl M.. (2019). Durum wheat genome highlights past domestication signatures and future improvement targets. Nat. Genet. 51, 885–895. doi: 10.1038/s41588-019-0381-3 30962619

[B110] Mamluk O. F. Nachit M. M. (1994). Sources of resistance to common bunt (*Tilletia foetida* and t. caries) in durum wheat. J. Phytopathol. 142, 122–130. doi: 10.1111/j.1439-0434.1994.tb04522.x

[B108] Marone D. Russo M. A. Mores A. Ficco D. B. Laidò G. Mastrangelo A. M.. (2021). Importance of landraces in cereal breeding for stress tolerance. Plants 10, 1267. doi: 10.3390/plants10071267 34206299 PMC8309184

[B112] Martínez-Moreno F. Ammar K. Solís I. (2022). Global changes in cultivated area and breeding activities of durum wheat from 1800 to date: a historical review. Agronomy 12, 1135. doi: 10.3390/agronomy12051135

[B111] Martínez-Moreno F. Solís I. Noguero D. Blanco A. Özberk İ. Nsarellah N.. (2020). Durum wheat in the Mediterranean rim: Historical evolution and genetic resources. Genet. Resour. 67, 1415–1436. doi: 10.1007/s10722-020-00913-8

[B1019] Martínez-Moreno F. Giraldo P. Cátedra M. D. M. Ruiz M. (2021). Evaluation of leaf rust resistance in the Spanish core collection of tetraploid wheat landraces and association with ecogeographical variables. Agriculture 11, 277. doi: 10.3390/agriculture11040277

[B109] Marzario S. Logozzo G. David J. L. Zeuli P. S. Gioia T. (2018). Molecular genotyping (SSR) and agronomic phenotyping for utilization of durum wheat (*Triticum durum* desf.) ex situ collection from southern Italy: a combined approach including pedigreed varieties. Genes 9, 465. doi: 10.3390/genes9100465 30241387 PMC6211131

[B113] Maucieri C. Caruso C. Bona S. Borin M. Barbera A. C. Cavallaro V. (2018). Influence of salinity and osmotic stress on germination process in an old sicilian landrace and a modern cultivar of *Triticum durum* desf. Cereal Res. Commun. 46, 253–262. doi: 10.1556/0806.46.2018.07

[B1020] Mazzucotelli E. Sciara G. Mastrangelo A. M. Desiderio F. Xu S. S. Faris J.. (2020). The global durum wheat panel (GDP): An international platform to identify and exchange beneficial alleles. Front. Plant Sci. 11. doi: 10.3389/fpls.2020.569905 PMC7779600 33408724

[B114] Medini M. Ferjaoui S. Bahri B. Mhri W. Hattab S. Hamza S. (2014). Bulk segregant analysis and marker-trait association reveal common AFLP markers for resistance to septoria leaf blotch in Tunisian old durum wheat. BASE.

[B1021] Mehrabi A. A. Steffenson B. J. Pour-Aboughadareh A. Matny O. Rahmatov M. (2022). Genome-wide association study identifies two loci for stripe rust resistance in a durum wheat panel from Iran 4963. doi: 10.3390/app12104963

[B115] Melini V. Melini F. Acquistucci R. (2021). Nutritional characterization of an Italian traditional bread from ancient grains: The case study of the durum wheat bread “Pane di monreale”. Eur. Food Res. Technol. 247 (1), 193–200. doi: 10.1007/s00217-020-03617-6

[B1022] Melnikova N. V. Mitrofanova O. P. Liapounova O. A. Kudryavtsev A. M. (2010). Global diversity of durum wheat *Triticum durum* desf. for alleles of gliadin-coding loci. Russ J. Genet. 46, 43–49. doi: 10.1134/S1022795410010072 20198879

[B116] Mengistu D. K. Kidane Y. G. Catellani M. Frascaroli E. Fadda C. Pè M. E.. (2016). High-density molecular characterization and association mapping in Ethiopian durum wheat landraces reveals high diversity and potential for wheat breeding. Plant Biotechnol. J. 14, 1800–1812. doi: 10.1111/pbi.12538 26853077 PMC5067613

[B117] Meyer R. S. Choi J. Y. Sanches M. Plessis A. Flowers J. M. Amas J.. (2016). Domestication history and geographical adaptation inferred from a SNP map of African rice. Nat. Genet. 48, 1083–1088. doi: 10.1038/ng.3633 27500524

[B118] Miazzi M. M. Babay E. De Vita P. Montemurro C. Chaabane R. Taranto F.. (2022). Comparative genetic analysis of durum wheat landraces and cultivars widespread in Tunisia. Front. Plant Sci. 13:939609. doi: 10.3389/fpls.2022.939609 35909756 PMC9326505

[B1023] Michel S. Kummer C. Gallee M. Hellinger J. Ametz C. Akgöl B.. (2018). Improving the baking quality of bread wheat by genomic selection in early generations. Theor. Appl. Genet. 131, 477–493. doi: 10.1007/s00122-017-2998-x 29063161 PMC5787228

[B120] Mohammadi R. Haghparast R. Sadeghzadeh B. Ahmadi H. Solimani K. Amri A. (2014). Adaptation patterns and yield stability of durum wheat landraces to highland cold rainfed areas of Iran. Crop Sci. 54, 944–954. doi: 10.2135/cropsci2013.05.0343

[B119] Mo Y. Howell T. Vasquez-Gross H. De Haro L. A. Dubcovsky J. Pearce S. (2018). Mapping causal mutations by exome sequencing in a wheat TILLING population: a tall mutant case study. Mol. Genet. Genomics 293, 463–477. doi: 10.1007/s00438-017-1401-6 29188438 PMC5854723

[B121] Moragues M. Zarco-Hernandez J. Moralejo M. A. Royo C. (2006). Genetic diversity of glutenin protein subunits composition in durum wheat landraces [*Triticum turgidum* ssp. *turgidum* convar. *durum* (Desf.) MacKey] from the Mediterranean basin. Genet. Resour. Crop Evol. 53 (5), 993–1002. doi: 10.1007/s10722-004-7367-3

[B122] Moreno-Amores J. Michel S. Miedaner T. Longin C. F. H. Buerstmayr H. (2020). Genomic predictions for fusarium head blight resistance in a diverse durum wheat panel: An effective incorporation of plant height and heading date as covariates. Euphytica 216, 1–19. doi: 10.1007/s10681-019-2551-x

[B123] Mulugeta B. Tesfaye K. Geleta M. Johansson E. Hailesilassie T. Hammenhag C.. (2022). Multivariate analyses of Ethiopian durum wheat revealed stable and high yielding genotypes. PloS One 17, 0273008. doi: 10.1371/journal.pone.0273008 PMC9385061 35976886

[B124] Munns R. Hare R. A. James R. A. Rebetzke G. J. (2000). Genetic variation for improving the salt tolerance of durum wheat. Aust. J. Agric. Res. 51, 69–74. doi: 10.1071/AR99057

[B125] Munns R. James R. A. Läuchli A. (2006). Approaches to increasing the salt tolerance of wheat and other cereals. J. Exp. Bot. 57, 1025–1043. doi: 10.1093/jxb/erj100 16510517

[B126] Muqaddasi Q. H. (2017). ““15k SNP chip data for spring and winter wheat [Data set],” in Plant genomics and phenomics research data repository (PGP) (Germany: IPK Gatersleben, Seeland OT Gatersleben, Corrensstraße 3).

[B127] Naranjo T. Cuñado N. Santos J. L. (2022). Assessing the heat tolerance of meiosis in spanish landraces of tetraploid wheat *Triticum turgidum* . Plants 11, 1661. doi: 10.3390/plants11131661 35807613 PMC9268776

[B129] Nazco R. Peña R. J. Ammar K. Villegas D. Crossa J. Moragues M.. (2014a). Variability in glutenin subunit composition of Mediterranean durum wheat germplasm and its relationship with gluten strength. J. Agric. Sci. 152 (3), 379–393. doi: 10.1017/S0021859613000117 24791017 PMC4003854

[B130] Nazco R. Peña R. J. Ammar K. Villegas D. Crossa J. Royo C. (2014b). Durum wheat (*Triticum durum* desf.) Mediterranean landraces as sources of variability for allelic combinations at glu-1/Glu-3 loci affecting gluten strength and pasta cooking quality. Genet. Resour. Crop Evol. 61, 1219–1236. doi: 10.1007/s10722-014-0104-7

[B128] Nazco R. Villegas D. Ammar K. Pena R. J. Moragues M. Royo C. (2012). Can Mediterranean durum wheat landraces contribute to improved grain quality attributes in modern cultivars? Euphytica 185 (1), 1–7. doi: 10.1007/s10681-011-0588-6

[B1024] Olivera P. D. Bulbula W. D. Badebo A. Bockelman H. E. Edae E. A. Jin Y. (2021). Field resistance to wheat stem rust in durum wheat accessions deposited at the USDA national small grains collection. Crop Sci. 61, 2565–2578. doi: 10.1002/csc2.20466 34413535 PMC8361663

[B131] Ostezan A. McDonald S. C. Tran D. T. Souza R. S. E. Li Z. (2021). Target region sequencing and applications in plants. JCSB 24, 13–26.

[B132] Ouaja M. Aouini L. Bahri B. Ferjaoui S. Medini M. Marcel T. C.. (2020). Identification of valuable sources of resistance to *Zymoseptoria tritici* in the Tunisian durum wheat landraces. Eur. J. Plant Pathol. 156, 647–661. doi: 10.1007/s10658-019-01914-9

[B133] Özkan H. Brandolini A. Schäfer-Pregl R. Salamini F. (2002). AFLP analysis of a collection of tetraploid wheats indicates the origin of emmer and hard wheat domestication in southeast Turkey. MBE 19, 1797–1801. doi: 10.1093/oxfordjournals.molbev.a004002 12270906

[B134] Palumbo M. Blangiforti S. Cambrea M. Gallo G. Licciardello S. Spina A. (2008). “Sicilian Durum wheat landraces for production of traditional breads,” in Proceedings of the International Durum Wheat Symposium “From seed to pasta: the durum wheat chain”, Bologna, Italy, 132.

[B135] Pavan S. Delvento C. Ricciardi L. Lotti C. Ciani E. D’Agostino N. (2020). Recommendations for choosing the genotyping method and best practices for quality control in crop genome-wide association studies. Front. Genet. 11, 447. doi: 10.3389/fgene.2020.00447 32587600 PMC7299185

[B136] Pecetti L. Boggini G. Gorham J. (1994). Performance of durum wheat landraces in a Mediterranean environment (eastern Sicily). Euphytica 80, 191–199. doi: 10.1007/BF00039650

[B137] Perry D. J. Lee S. J. (2017). Durum wheat variety identification by OpenArray analysis. Can. J. Plant Sci. 97, 403–407. doi: 10.1139/cjps-2016-0300

[B138] Poland J. Endelman J. Dawson J. Rutkoski J. Wu S. Manes Y.. (2012). Genomic selection in wheat breeding using genotyping-by-sequencing. TPG 5. doi: 10.3835/plantgenome2012.06.0006

[B139] Qureshi N. Bariana H. Kolmer J. A. Miah H. Bansal U. (2017). Genetic and molecular characterization of leaf rust resistance in two durum wheat landraces. Phytopathol 107, 1381–1387. doi: 10.1094/PHYTO-01-17-0005-R 28812937

[B140] Qureshi N. Bariana H. Kumran V. V. Muruga S. Forrest K. L. Hayden M. J.. (2018). A new leaf rust resistance gene *Lr79* mapped in chromosome 3BL from the durum wheat landrace Aus26582. Theor. App. Genet. 131, 1091–1098. doi: 10.1007/s00122-018-3060-3 29396589

[B142] Rasheed A. Mujeeb-Kazi A. Ogbonnaya F. C. He Z. H. Rajaram S. (2018). Wheat genetic resources in the post-genomics era: promise and challenges. Ann. Bot-London 121, 603–616. doi: 10.1093/aob/mcx148 PMC5852999 29240874

[B141] Rasheed A. Xia X. (2019). From markers to genome-based breeding in wheat. Theor. App. Genet. 132, 767–784. doi: 10.1007/s00122-019-03286-4 30673804

[B143] Rellstab C. Gugerli F. Eckert A. J. Hancock A. M. Holderegger R. (2015). A practical guide to environmental association analysis in landscape genomics. Mol. Ecol. 24, 4348–4370. doi: 10.1111/mec.13322 26184487

[B144] Requena-Ramírez M. D. Hornero-Méndez D. Rodríguez-Suárez C. Atienza S. G. (2021). Durum wheat (*Triticum durum* l.) landraces reveal potential for the improvement of grain carotenoid esterification in breeding programs. Foods 10, 757. doi: 10.3390/foods10040757 33918139 PMC8067221

[B145] Requena-Ramírez M. D. Rodríguez-Suárez C. Flores F. Hornero-Méndez D. Atienza S. G. (2022). Marker-trait associations for total carotenoid content and individual carotenoids in durum wheat identified by genome-wide association analysis. Plants 11, 2065. doi: 10.3390/plants11152065 35956543 PMC9370666

[B146] Reynolds M. P. Hobbs P. R. Braun H. J. (2007). Challenges to international wheat improvement. J. Agric. Sci. 145, 223. doi: 10.1017/S0021859607007034

[B147] Reynolds M. P. Lewis J. M. Ammar K. Basnet B. R. Crespo-Herrera L. Crossa J.. (2021). Harnessing translational research in wheat for climate resilience. JXB 72, 5134–5157. doi: 10.1093/jxb/erab256 PMC8272565 34139769

[B148] Roberts D. R. Bahn V. Ciuti S. Boyce M. S. Elith J. Guillera-Arroita G.. (2017). Cross-validation strategies for data with temporal, spatial, hierarchical, or phylogenetic structure. Ecography 40, 913–929. doi: 10.1111/ecog.02881

[B150] Roncallo P. F. Guzmán C. Larsen A. O. Achilli A. L. Dreisigacker S. Molfese E.. (2021). Allelic variation at glutenin loci (*Glu-1*, glu-2 and *Glu-3*) in a worldwide durum wheat collection and its effect on quality attributes. Foods 11, 2845. doi: 10.3390/foods10112845 PMC8623136 34829126

[B151] Roselló M. Royo C. Álvaro F. Villegas D. Nazco R. Soriano J. M. (2018). Pasta-making quality QTLome from Mediterranean durum wheat landraces. Front. Plant Sci. 9, 1512. doi: 10.3389/fpls.2018.01512 30459781 PMC6232839

[B152] Royo C. Nazco R. Villegas D. (2014). The climate of the zone of origin of Mediterranean durum wheat (*Triticum durum* desf.) landraces affects their agronomic performance. Genet. Resour. 61, 1345–1358. doi: 10.1007/s10722-014-0116-3

[B1025] Royo C. Ammar K. Villegas D. Soriano J. M. (2021). Agronomic, physiological and genetic changes associated with evolution, migration and modern breeding in durum wheat. Front. Plant Sci. 12. doi: 10.3389/fpls.2021.674470 PMC8296143 34305973

[B2000] Royo C. Dreisigacker S. Soriano J. M. Lopes M. S. Ammar K. Villegas D. (2020). Allelic variation at the vernalization response (Vrn-1) and photoperiod sensitivity (Ppd-1) genes and their association with the development of durum wheat landraces and modern cultivars. Front. Plant Sci. 11–838. 32655598 10.3389/fpls.2020.00838 PMC7325763

[B153] Ruisi P. Ingraffia R. Urso V. Giambalvo D. Alfonzo A. Corona O.. (2021). Influence of grain quality, semolinas and baker’s yeast on bread made from old landraces and modern genotypes of Sicilian durum wheat. Int. Food Res. J. 140, 110029. doi: 10.1016/j.foodres.2020.110029 33648257

[B155] Ruiz M. Bernal G. Giraldo P. (2018). An update of low molecular weight glutenin subunits in durum wheat relevant to breeding for quality. J. Cereal Sci. 83, 236–244. doi: 10.1016/j.jcs.2018.09.005

[B154] Ruiz M. Giraldo P. Royo C. Villegas D. Aranzana M. J. Carrillo J. M. (2012). Diversity and genetic structure of a collection of Spanish durum wheat landraces. Crop Sci. 52, 2262–2275. doi: 10.2135/cropsci2012.02.0081

[B1026] Saccomanno A. Matny O. Marone D. Laidò G. Petruzzino G. Mazzucotelli E.. (2018). Genetic mapping of loci for resistance to stem rust in a tetraploid wheat collection. Int. J. Mol. Sci. 19, 3907. doi: 10.3390/ijms19123907 30563213 PMC6321032

[B156] Sahri A. Chentoufi L. Arbaoui M. Ardisson M. Belqadi L. Birouk A.. (2014). Towards a comprehensive characterization of durum wheat landraces in Moroccan traditional agrosystems: analysing genetic diversity in the light of geography, farmers’ taxonomy and tetraploid wheat domestication history. BMC evol. Biol. 14, 1–18. doi: 10.1186/s12862-014-0264-2 PMC4300848 25528060

[B157] Saintenac C. Jiang D. Akhunov E. D. (2011). Targeted analysis of nucleotide and copy number variation by exon capture in allotetraploid wheat genome. Genome Biol. 12, R88. doi: 10.1186/gb-2011-12-9-r88 21917144 PMC3308051

[B158] Sandve S. R. Rudi H. Dørum G. Vigeland M. D. Berg P. R. Rognli O. A. (2010). “Genotyping unknown genomic terrain in complex plant genomes,” in Sustainable use of genetic diversity in forage and turf breeding. Ed. Huyghe C. (New Mexico: Springer), 455–459.

[B159] Sareen S. Bhusal N. Kumar M. Bhati P. K. Munjal R. Kumari J.. (2020). Molecular genetic diversity analysis for heat tolerance of indigenous and exotic wheat genotypes. J. Plant Biochem. Biotechnol. 29, 15–23. doi: 10.1007/s13562-019-00501-7

[B160] Scarascia Mugnozza G. T. (2005). ‘The contribution of Italian wheat geneticists: from Nazareno Strampelli to Francesco D'Amato’. Rome: Accademia Nazionale delle Scienze, 53–75.

[B161] Scavo A. Pandino G. Restuccia A. Caruso P. Lombardo S. Mauromicale G. (2022). Allelopathy in durum wheat landraces as affected by genotype and plant part. Plants 11, 1021. doi: 10.3390/plants11081021 35448748 PMC9026900

[B162] Scheben A. Batley J. Edwards D. (2018). Revolution in genotyping platforms for crop improvement. Plant Genet. Mol. Biol. 164, 37–52. doi: 10.1007/10_2017_47 29356847

[B163] Schulthess A. W. Kale S. M. Liu F. Zhao Y. Philipp N. Rembe M.. (2022). Genomics-informed prebreeding unlocks the diversity in genebanks for wheat improvement. Nat. Genet. 54, 1544–1552. doi: 10.1038/s41588-022-01189-7 36195758

[B166] Shamaya N. J. Shavrukov Y. Langridge P. Roy S. J. Tester M. (2017). Genetics of na+ exclusion and salinity tolerance in Afghani durum wheat landraces. BMC Plant Biol. 17, 1–8. doi: 10.1186/s12870-017-1164-6 29157217 PMC5697363

[B167] Shan D. Ali M. Shahid M. Arif A. Waheed M. Q. Xia X.. (2022). Genetic networks underlying salinity tolerance in wheat uncovered with genome-wide analyses and selective sweeps. Theor. Appl. Genet. 135, 2925–2941. doi: 10.1007/s00122-022-04153-5 35915266

[B164] Sharma S. Schulthess A. W. Bassi F. M. Badaeva E. D. Neumann K. Graner A.. (2021). Introducing beneficial alleles from plant genetic resources into the wheat germplasm. Biology 10, 982. doi: 10.3390/biology10100982 34681081 PMC8533267

[B165] Shavrukov Y. Shamaya N. Baho M. Edwards J. Ramsey C. Nevo E.. (2011). Salinity tolerance and na+ exclusion in wheat: variability, genetics, mapping populations and QTL analysis. Czech J. Genet. Plant Breed 47, 85–93. doi: 10.17221/3260-CJGPB

[B168] Shayanmehr S. Henneberry S. R. Sabouhi M. S. Foroushani N. S. (2020). Drought, climate change, and dryland wheat yield response: An econometric approach. Int. J. Environ. Res. Public Health 17, 5264. doi: 10.3390/ijerph17145264 32708323 PMC7399810

[B169] Shewry P. (2019). What is gluten–why is it special? Front. Nutr. 101. doi: 10.3389/fnut.2019.00101 PMC6625226 31334243

[B170] Soriano J. M. Villegas D. Sorrells M. E. Royo C. (2018). Durum wheat landraces from east and west regions of the mediterranean basin are genetically distinct for yield components and phenology. Front. Plant Sci. 9, 80. doi: 10.3389/fpls.2018.00080 29472936 PMC5809869

[B171] Sork V. L. Aitken S. N. Dyer R. J. Eckert A. J. Legendre P. Neale D. B. (2013). Putting the landscape into the genomics of trees: Approaches for understanding local adaptation and population responses to changing climate. Tree Genet. Genomes 9, 901–911. doi: 10.1007/s11295-013-0596-x

[B1027] Soriano J. M. Colasuonno P. Marcotuli I. Gadaleta A. (2021). Meta-QTL analysis and identification of candidate genes for quality, abiotic and biotic stress in durum wheat. Sci. Rep. 11, 11877. doi: 10.1038/s41598-021-91446-2 34088972 PMC8178383

[B172] Spina A. Dinelli G. Palumbo M. Whittaker A. Cambrea M. Negri L.. (2021). Evaluation of standard physico-chemical and rheological parameters in predicting bread-making quality of durum wheat (*Triticum turgidum* l. ssp. *durum* [Desf.] husn.). Int. J. Food Sci. 56, 3278–3288. doi: 10.1111/ijfs.15018

[B173] Spinoni J. Vogt J. V. Naumann G. Barbosa P. Dosio A. (2018). Will drought events become more frequent and severe in Europe? Int. J. Climatol. 38, 1718–1736. doi: 10.1002/joc.5291

[B174] Steemers F. J. Gunderson K. L. (2007). Whole genome genotyping technologies on the BeadArray™ platform. Biotechnol. Journal: Healthcare Nutr. Technol. 2, 41–49. doi: 10.1002/biot.200600213 17225249

[B175] Subira J. Peña R. J. Álvaro F. Ammar K. Ramdani A. Royo C. (2014). Breeding progress in the pasta-making quality of durum wheat cultivars released in Italy and Spain during the 20th century. Crop Pasture Sci. 65 (1), 16–26. doi: 10.1071/CP13238

[B176] Sukumaran S. Reynolds M. P. Sansaloni C. (2018). Genome-wide association analyses identify QTL hotspots for yield and component traits in durum wheat grown under yield potential, drought, and heat stress environments. Front. Plant Sci. 9, 81. doi: 10.3389/fpls.2018.00081 29467776 PMC5808252

[B177] Taghouti M. Rhrib K. Gaboun F. (2013). “Exploiting landrace genetic diversity for germplasm enhancement in durum wheat breeding in Morocco,” in: Porceddu E. Damania A. B. Qualset C. O. (ed.). Proceedings of the International Symposium on Genetics and Breeding of Durum Wheat. Bari: CIHEAM. p. 109–119 (Options Méditerranéennes : Série A. Séminaires Méditerranéens; n. 110)

[B178] Talas F. Longin F. Miedaner T. (2011). Sources of resistance to fusarium head blight within Syrian durum wheat landraces. Plant Breed. 130, 398–400. doi: 10.1111/j.1439-0523.2011.01867.x

[B179] Tan A. (2002). In situ on-farm conservation of landraces grown in north-Western transitional zone of Turkey (in Turkish), Sonuc Raporu (final report). Tubitak-Togtag-2347. Tübitak, Ankara.

[B180] Tanksley S. D. McCouch S. R. (1997). Seed banks and molecular maps: unlocking genetic potential from the wild. Science 277, 1063–1066. doi: 10.1126/science.277.5329.1063 9262467

[B183] Taranto F. D’Agostino N. Rodriguez M. Pavan S. Minervini A. P. Pecchioni N.. (2020). Whole genome scan reveals molecular signatures of divergence and selection related to important traits in durum wheat germplasm. Front. Genet. 11, 217. doi: 10.3389/fgene.2020.00217 32373150 PMC7187681

[B184] Taranto F. Di Serio E. Miazzi M. M. Pavan S. Saia S. De Vita P.. (2022). Intra-and inter-population genetic diversity of “Russello” and “Timilia” landraces from Sicily: A proxy towards the identification of favorable alleles in durum wheat. Agronomy 12, 1326. doi: 10.3390/agronomy12061326

[B181] Taranto F. Mangini G. Pasqualone A. Gadaleta A. Blanco A. (2015). Mapping and allelic variations of *Ppo-B1* and *Ppo-B2* gene-related polyphenol oxidase activity in durum wheat. Mol. Breed. 35 (2), 1–10. doi: 10.1007/s11032-015-0272-y

[B182] Taranto F. Nicolia A. Pavan S. De Vita P. D’Agostino N. (2018). Biotechnological and digital revolution for climate-smart plant breeding. Agronomy 8 (12), 277. doi: 10.3390/agronomy8120277

[B185] Thomson M. J. (2014). High-throughput SNP genotyping to accelerate crop improvement. Plant Breed. Biotech. 2, 195–212. doi: 10.9787/PBB.2014.2.3.195

[B186] Tibbs Cortes L. Zhang Z. Yu J. (2021). Status and prospects of genome-wide association studies in plants. TPG 14, 20077. doi: 10.1002/tpg2.20077 PMC12806871 33442955

[B187] Trebbi D. Maccaferri M. de Heer P. Sørensen A. Giuliani S. Salvi S.. (2011). High-throughput SNP discovery and genotyping in durum wheat (*Triticum durum* desf.). Theor. Appl. Genet. 123, 555–569. doi: 10.1007/s00122-011-1607-7 21611761

[B188] Van Orsouw N. J. Hogers R. C. Janssen A. Yalcin F. Snoeijers S. Verstege E.. (2007). Complexity reduction of polymorphic sequences (CRoPS™): a novel approach for large-scale polymorphism discovery in complex genomes. PloS One 2 (11), e1172. doi: 10.1371/journal.pone.0001172 18000544 PMC2048665

[B1028] Varella A. C. Zhang H. Weaver D. K. Cook J. P. Hofland M. L.. (2019). A novel QTL in durum wheat for resistance to the wheat stem sawfly associated with early expression of stem solidness. G3 (Bethesda). 9, 1999–2006. doi: 10.1534/g3.119.400240 31015195 PMC6553545

[B189] Visioli G. Giannelli G. Agrimonti C. Spina A. Pasini G. (2021). Traceability of Sicilian durum wheat landraces and historical varieties by high molecular weight glutenins footprint. Agronomy 11 (1), 143. doi: 10.3390/agronomy11010143

[B190] Vita F. Taiti C. Pompeiano A. Gu Z. Lo Presti E. Whitney L.. (2016). Aromatic and proteomic analyses corroborate the distinction between Mediterranean landraces and modern varieties of durum wheat. Sci. Rep. 6, 1–15. doi: 10.1038/srep34619 27708424 PMC5052571

[B192] Wang Z. Wang W. Xie X. Wang Y. Yang Z. Peng H.. (2022). Dispersed emergence and protracted domestication of polyploid wheat uncovered by mosaic ancestral haploblock inference. Nat. Commun. 13, 1–14. doi: 10.1038/s41467-022-31581-0 35794156 PMC9259585

[B193] Wang S. Wong D. Forrest K. Allen A. Chao S. Huang B. E.. (2014). Characterization of polyploid wheat genomic diversity using a high-density 90 000 single nucleotide polymorphism array. Plant Biotechnol. J. 12, 787–796. doi: 10.1111/pbi.12183 24646323 PMC4265271

[B191] Wang S. Xu S. Chao S. Sun Q. Liu S. Xia G. (2019). A genome-wide association study of highly heritable agronomic traits in durum wheat. Front. Plant Sci. 10, 919. doi: 10.3389/fpls.2019.00919 31379901 PMC6652809

[B196] Watson A. Ghosh S. Williams M. J. Cuddy W. S. Simmonds J. Rey M. D.. (2018). Speed breeding is a powerful tool to accelerate crop research and breeding. Nat. Plants 4 (1), 23–29. doi: 10.1038/s41477-017-0083-8 29292376

[B194] Winfield M. O. Allen A. M. Burridge A. J. Barker G. L. Benbow H. R. Wilkinson P. A.. (2016). High-density SNP genotyping array for hexaploid wheat and its secondary and tertiary gene pool. Plant Biotechnol. J. 14, 1195–1206. doi: 10.1111/pbi.12485 26466852 PMC4950041

[B195] Xynias I. N. Mylonas I. Korpetis E. G. Ninou E. Tsaballa A. Avdikos I. D.. (2020). Durum wheat breeding in the Mediterranean region: Current status and future prospects. Agronomy 10, 432. doi: 10.3390/agronomy10030432

[B199] Zeven A. C. (1999). The traditional inexplicable replacement of seed and seed ware of landraces and cultivars: a review. Euphytica 110, 181–191. doi: 10.1023/A:1003701529155

[B198] Zhou Z. Jiang Y. Wang Z. Gou Z. Lyu J. Li W.. (2015). Resequencing 302 wild and cultivated accessions identifies genes related to domestication and improvement in soybean. Nat. Biotechnol. 33 (4), 408–414. doi: 10.1038/nbt.3096 25643055

[B197] Zhou X. Stephens M. (2012). Genome-wide efficient mixed model analysis for association studies. Nat. Genet. 44, 821–824. doi: 10.1038/ng.2310 22706312 PMC3386377

